# The acute phase protein lactoferrin is a key feature of Alzheimer’s disease and predictor of Aβ burden through induction of APP amyloidogenic processing

**DOI:** 10.1038/s41380-021-01248-1

**Published:** 2021-08-16

**Authors:** Andrew Tsatsanis, Andrew N. McCorkindale, Bruce X. Wong, Ellis Patrick, Tim M. Ryan, Robert W. Evans, Ashley I. Bush, Greg T. Sutherland, Asipu Sivaprasadarao, Boris Guennewig, James A. Duce

**Affiliations:** 1 The ALBORADA Drug Discovery Institute, University of Cambridge, Cambridge Biomedical Campus, Cambridge, UK; 2 Faculty of Biological Sciences, School of Biomedical Sciences, University of Leeds, Leeds, West Yorkshire UK; 3 Faculty of Medicine and Health, Charles Perkins Centre and School of Medical Sciences, University of Sydney, Camperdown, NSW Australia; 4 Melbourne Dementia Research Centre, The Florey Institute of Neuroscience and Mental Health, The University of Melbourne, Melbourne, VIC Australia; 5 Faculty of Science, School of Mathematics and Statistics, University of Sydney, Camperdown, NSW Australia; 6 School of Engineering and Design, Brunel University, London, UK; 7 Faculty of Medicine and Health, Brain and Mind Centre and School of Medical Sciences, The University of Sydney, Camperdown, NSW Australia

**Keywords:** Neuroscience, Cell biology

## Abstract

Amyloidogenic processing of the amyloid precursor protein (APP) forms the amyloid-β peptide (Aβ) component of pathognomonic extracellular plaques of AD. Additional early cortical changes in AD include neuroinflammation and elevated iron levels. Activation of the innate immune system in the brain is a neuroprotective response to infection; however, persistent neuroinflammation is linked to AD neuropathology by uncertain mechanisms. Non-parametric machine learning analysis on transcriptomic data from a large neuropathologically characterised patient cohort revealed the acute phase protein lactoferrin (Lf) as the key predictor of amyloid pathology. In vitro studies showed that an interaction between APP and the iron-bound form of Lf secreted from activated microglia diverted neuronal APP endocytosis from the canonical clathrin-dependent pathway to one requiring ADP ribosylation factor 6 trafficking. By rerouting APP recycling to the Rab11-positive compartment for amyloidogenic processing, Lf dramatically increased neuronal Aβ production. Lf emerges as a novel pharmacological target for AD that not only modulates APP processing but provides a link between Aβ production, neuroinflammation and iron dysregulation.

## Introduction

Alzheimer’s disease (AD) is a heterogenous disease with a complex aetiology. Deposition of amyloid-β peptide (Aβ) as plaques remains a primary neuropathological criterion for AD diagnosis despite the recent doubt drawn on the amyloid hypothesis in AD. What initiates the production and accumulation of Aβ in sporadic AD cases remains unresolved. Identifying novel underlying cellular and molecular processes critical in AD pathology may provide a clue as to how Aβ is implicated in the broader pathobiology of the disease.

Amyloid precursor protein (APP) is a homogenously expressed transmembrane protein and precursor to the proteolytically cleaved Aβ that commonly accumulates in the aged brain [1]. Full-length APP and proteolytic products other than Aβ have neurotrophic properties [2, 3], which include stabilising the iron export pore protein ferroportin on the cell surface to facilitate iron efflux [4, 5]. A functional role for Aβ remains to be elucidated despite evidence that the peptide is important for innate immunity through its antimicrobial properties [6–8].

As a semi-immune privileged region within the body, the brain may have evolved systems to minimise inflammation while protecting against infectious insult. Some hypothesise that this system becomes overwhelmed with aging and then contributes to neuropathogenesis [9, 10]. Along with Aβ plaques and tangle-bearing neurons, AD neuropathology is also characterised by reactive gliosis that is suspected of producing a neurotoxic milieu that contributes to neuronal degeneration [11, 12]. Microglial activation in AD and its mouse models appears to be Aβ dependent, mediated by binding to the NLR Family Pyrin Domain Containing 3 [13] as well as pattern recognition receptors such as RAGE, scavenger receptors [14] and toll‐like receptors (TLR2, TLR4 and TLR6) [15]. Proinflammatory damage by activated microglia is thought to occur early in AD pathogenesis [16] and may contribute to the APOE-e4 allele being a major risk for sporadic AD [17].

In an attempt to elucidate the pathogenic events in AD, transcriptomic analysis of AD-affected tissue has produced discordant results using the conventional differential expression analysis (DEA) [18]. This may reflect the lack of sufficiently stringent methods for both the processing and analysis of transcriptomic data combined with the difficulty of extracting reliable signal from high-dimensional data [19, 20]. Furthermore, DEA employs a parametric approach that is vulnerable to outliers, does not capture gene interactions and often produces thousands of differentially expressed genes (DEGs) [21, 22]. Therefore, we chose to interrogate transcriptomic data from AD tissue by applying random-forest-based machine learning algorithms, which employ a multivariate non-parametric approach that potentially detects genes missed by DEA [23]. Random-forest-based algorithms capture gene interactions, are robust to outliers and only select the features that improve model performance [24]. Boruta is a particularly robust and powerful feature selection method for extracting the key factors discovered with these algorithms [25].

RNA-seq data from the Religious Orders Study (ROS) and the Rush Memory and Aging Project (MAP) cohort have been analysed previously [26–28] but not to our knowledge using machine learning. More advanced RNA-seq bioinformatic tools such as the STAR aligner have become available since the data were first processed using the Bowtie aligner [29, 30]. Upon re-processing and machine learning analysis of the ROSMAP data in the present study, transcriptionally elevated *lactoferrin* (*LTF*) emerged as the most important feature, consistent with reports of histopathological elevation in protein levels of lactoferrin (Lf) with AD [31–34]. Lf has a limited role in normal extracellular iron transport, but its secretion into biofluids as part of an innate immune response suggests a requirement for it to sequester iron from invading pathogens [35, 36]. This acute phase protein also participates in a number of related physiological functions involving growth and differentiation [37], host defence against microbial infections [38] and anti-inflammatory protection against carcinogenesis [39]. This broad range of additional properties occurs through interactions with various intracellular proteins [40], secretory proteins [41] and receptors on cell surfaces [38]. Here, we test whether Lf could impact on AD pathobiology through the canonical Aβ generation pathway.

## Results

### Machine learning classification of ROSMAP cohort

**Fig. 1:**
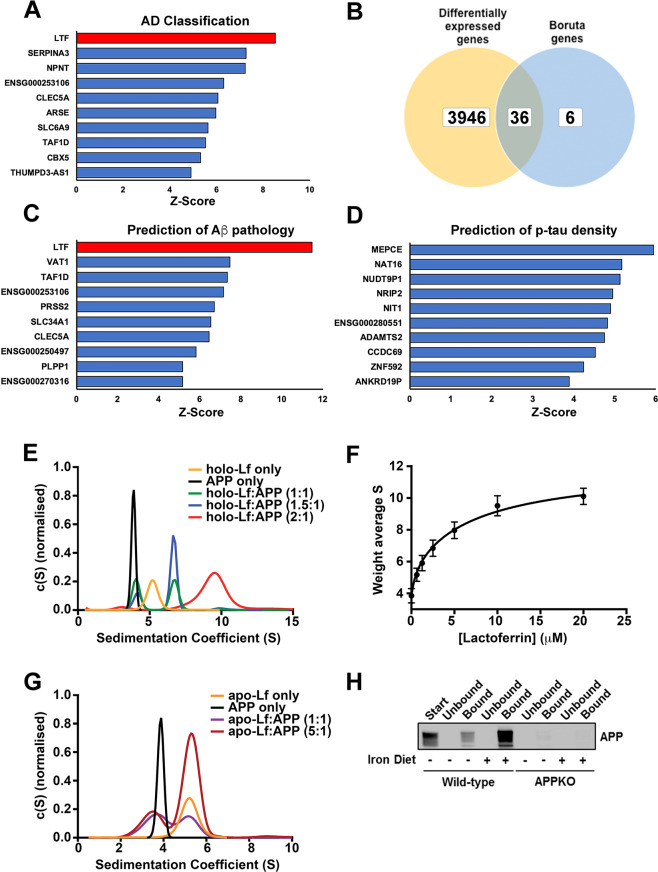
A biophysical interaction between APP and Lf supports machine learning analysis of a lead hit in classifying disease and predicting Aβ pathology. **A**, **B** The top 10 genes identified by *z*-score using the feature selection algorithm, Boruta for classifying the ROSMAP cohort into neuropathologically positive AD cases and controls (**A**). Of these genes a small fraction were not identified by differential expression analysis using EdgeR (**B**). **C** Top 10 genes ranked by *z*-score predicting total amount of amyloid pathology. **D** Top 10 genes ranked by *z*-score predicting p-tau (AT8) immunoreactivity across all seven cortical regions and the hippocampus. **E** Sedimentation coefficient distributions of holo-Lf alone (5 μM; orange) and APP (2.5 μM) in the absence (black) and presence of Lf (green: 2.5 μM; blue: 3.75 μM; red: 5 μM). The c(S) distributions indicate two different complexes that form in a concentration dependent manner. See Supplementary Fig. S2 for example data sets showing data quality and the fit of the c(s) model to the data. **F** The weight average sedimentation coefficient obtained by integrating the c(S) distribution (as shown in **E**) calculated as a function of Lf concentration (Eq 1 in Supplementary data) with the assumption that APP contains two binding sites for Lf with two different dissociation constants. **G** Sedimentation velocity analysis of apo-Lf alone (2.5 μM; orange) and APP (2.5 μM) in the absence (black) and presence of apo-Lf (purple: 2.5 μM; maroon: 12.5 μM). **H** Confirmation of an interaction of APP with Lf in vivo using anti-APP (1:1000; 22C11) for detection and anti-Lf (1:200) for immunoprecipitation of brain homogenates from APP–/– mice and littermate controls either on a normal or high-iron diet. Specificity of interaction of APP to Lf was confirmed using anti-β-actin as the capture antibody (data not shown). Data are mean ± SEM of three experiments performed in triplicate.

The ROSMAP RNA-seq data were investigated using machine learning, including the Boruta feature selection algorithm. As the Boruta algorithm ranks genes by *Z*-score, 42 of 23,056 genes were designated as important in classifying post-mortem cortical histopathology as AD or control (NIA-Reagan criteria). The *LTF* gene was ranked highest (Fig.1A and Supplementary Table S1A) being 8.53 standard deviations more important than the mean feature (for *Z*-score of 8.53 the cumulative probability = 0.999 and *q* value = 1/∞). The AD versus control classification analysis was then repeated with four algorithms using repeated cross-fold validation. *LTF* was the top gene in the ‘random forest’ and ‘rpart’ models, second in the ‘xgbtree’ and 80th in the ‘glmnet’ model (see Supplementary Table S2 in Supplementary File for top 20 ranked genes for each algorithm).

The more conventional DEA using EdgeR was additionally applied to the same gene set. This identified 3982 DEGs between the AD and control groups. Of the 42 genes identified by Boruta, 36 were also DEGs (Fig. 1B and Supplementary Table S1A) including 4 (*SLC6A9*, *SLC4A11*, *C1QTNF5* and *LINC02393*) that were among the top 10 DEGs when ranked by false discovery rate (Supplementary Table S3). Despite having the 35th greatest fold change of all DEGs, *LTF* was only determined to be modestly upregulated in AD (a 0.5338 log2 fold change representing a 44.8%. increase) and ranked 2360th overall. The low ranking in the DEA compared to Boruta is likely due to negligible *LTF* expression in the majority of controls (the top four control subjects accounted for 55% of the total control *LTF* expression). The EdgeR results were validated by running another DEA using DESeq2. There were 3980 DEGs with 3778 common (~95%) to the EdgeR DEGs and *LTF* again ranked lowly at 1617th. Although no enriched pathways were apparent among the 42 genes identified by Boruta using the STRING or WebGestalt databases, Gene Ontology (GO) analysis using the WebGestalt database did identify 199 significantly enriched categories from the DEA, of which 16 of the top 20 significantly enriched categories were metabolic processes (Supplementary Table S4). Similarly, a Kyoto Encyclopaedia of Genes and Genomes pathway analysis using WebGestalt identified 15 significantly enriched pathways with ‘Oxidative Phosphorylation’ being the most enriched (Supplementary Table S5). The DEGs were compared against the Open Targets platform list of genes with an RNA expression association to AD (*n* = 3199). This identified 943 of the DEGs present (including *LTF*) (Supplementary Table S6) of which 3 were also top 10 DEGs (*SLC4A11*, *S100A4* and *ADAMTS2*).

The Boruta algorithm was then used to identify genes that predicted the quantity of AD pathology in each individual. Regression analysis was done on total areal fraction of immunopositive Aβ and cortical density of abnormally phosphorylated tau at serine 202 (p-tau) across all quantified brain regions (comprising seven cortical regions and the hippocampus) as well as the superior frontal gyrus (SFG); the region adjacent to where the RNA-seq samples were from. There were 30 genes predicting total Aβ (Fig. 1C and Supplementary Table S1B) and 17 that predicted Aβ within the SFG (Supplementary Fig. S1A and Supplementary Table S1C), of which 4 were in common (*LTF*, *VAT1*, *PRSS2* and *AC090198.1*). *LTF* was highest ranked for total and SFG Aβ. For p-tau predictions, 16 were identified from all regions (Fig. 1D and Supplementary Table S1D) and 5 within the SFG (Supplementary Table S1E); the power of analysis in the latter being limited by 192 subjects lacking tau data or having no expression. The only gene common at predicting p-tau in all regions was *ENSG00000280551*, a gene of unknown role. *LTF* and *TAF1D* were the only two genes that were predictive of both Aβ and p-tau in all quantified brain regions but *LTF* was only ranked 15th for p-tau.

GO biological process analysis using the STRING database indicated an enrichment of genes from the ‘neutrophil degranulation’ and ‘localisation’ pathways that predicted total Aβ and three bone development pathways (GO#s 1900157, 1903011 and 0048705) that predicted total p-tau. For total Aβ, this was in part supported by an enrichment for genes from the REACTOME ‘neutrophil degranulation’ pathway with both gene lists (seven genes for all-cortical Aβ prediction and four for SFG Aβ prediction). *LTF*, *VAT1* and *AC090198.1* were also present in the 42 genes identified by disease classification analysis.

Although *LTF* has not been ranked highly by DEA here or previously, it is consistently upregulated in AD including both the Open Targets [42, 43] and AD consensus transcriptomics [44] platforms. The latter is a combined analysis of the ROSMAP (as used here), Mayo Clinic AD and Mount Sinai School of Medicine (MSSM) RNA-seq datasets with a further three microarray validation datasets. *LTF* expression was significantly higher in the Mayo dataset (*p* < 0.0001) (Supplementary Fig. S1Bi), trended higher in the MSSM frontal pole dataset (Supplementary Fig. S1Bii) and was higher in all microarray datasets (in particular the larger dataset from Zhang et al.; *p* < 0.001) (Supplementary Fig. S1C). As *LTF* appeared to be upregulated in AD and strongly predictive of Aβ but not tau, we proceeded to investigate the possible interactions of Lf protein with the canonical biochemistry of Aβ generation.

### In vitro and in vivo identification of an interaction between APP and Lf

We initially determined whether recombinant α-secretase N-terminal soluble form of APP695 (sAPPα^695^) bound to recombinant holo-Lf. Alone, both sAPPα^695^ and holo-Lf produced a single homogenous Gaussian distribution peak (c(S)) upon sedimentation velocity analysis, with modal sedimentation coefficients of 3.9 S (*f*/*f*0 = 1.40, MW ≈ 61 kDa) and 5.1 S (*f*/*f*0 = 1.38, MW ≈ 79 kDa), respectively (Fig. 1E and Supplementary Fig. S2A, B). Mixing of sAPPα^695^ with holo-Lf at molar ratios of 1:1 resulted in a bimodal distribution with maximal sedimentation coefficients at ~4 S and 6.9 S (*f*/*f*0 = 1.39, MW ≈ 61 and 142 kDa) (Fig. 1E and Supplementary Fig. S2C, D). The significant shift in the second c(S) to a higher sedimentation coefficient was indicative of a strong interaction. Increasing the protein complex ratio to 1.5:1 and 2:1 (holo-Lf:sAPPα^695^) resulted in the emergence of a third c(S) distribution peak at ≈9.8 S (*f*/*f*0 = 1.36, MW ≈ 223 kDa) with the disappearance of the lighter peaks (Fig. 1E), consistent with a stoichiometry of two Lf molecules per sAPPα^695^ molecule. Integrating these c(S) distributions enabled weight average sedimentation coefficients (Fig. 1F) to be fitted to a two-site binding model for obtaining estimates of Kd_1:1_ 620 nM and Kd_1:2_ 8.2 µM (Supplementary Table S7; *R*^2^ = 0.98). By contrast, fitting to single site models was poor, with a non-random distribution of residuals (data not shown; *R*^2^ < 0.92). In the presence of equimolar holo-Lf, alternative isoforms of APP (sAPPα^751^ and sAPPα^770^) produced similar bimodal c(S) distributions (Supplementary Fig. S2D) indicating that the additional domains present in these larger isoforms did not alter the interaction with Lf. Mixing apo-Lf (with a very similar sedimentation distribution to holo-Lf) and sAPPα^695^ at a 1:1 or 5:1 molar ratio resulted in bimodal sedimentation coefficients (3.95 S and 5.0 S) (Fig. 1G) corresponding to values of APP and apo-Lf alone. This indicates that Lf must be iron-bound to interact with APP.

In support, a 1.15-fold increase in native tryptophan fluorescence from sAPPα^695^ in the presence of excess holo-Lf was observed in comparison to the simple addition of the spectra of these two macromolecules (Supplementary Fig. S2E). Furthermore, while the spectral maxima of the two proteins are not significantly different, the spectrum of the mixture is sharper in the longer wavelength region than that of the addition spectra of the two macromolecules (Supplementary Fig. S2F), indicating a blue shift in one or more of the 15 tryptophan residues present in these macromolecules. The increased fluorescence intensity and sharpening of the spectrum both suggest that at least one tryptophan is being protected from solvent as part of this interaction. The change in fluorescence intensity as a function of Lf concentration (Supplementary Fig. S2G) was a better fit to a two-site model (*R*^2^ = 0.99) compared to a single site model (*R*^2^ of 0.97), providing Kd_1:1_ 690 nM and Kd_1:2_ 9.4 µM estimates in accord with the sedimentation analysis (Supplementary Table S7). No consistent change in tryptophan fluorescence was observed upon mixing either apo-Lf or either form of holo- or apo-transferrin (Tf) with sAPPα^695^.

To determine whether such an interaction between APP and Lf occurred in vivo, Lf binding partners were immunoprecipitated from mouse brain extracts (Fig. 1H). APP prominently co-eluted with Lf, intensity of which was found to be markedly greater with brain extracts derived from mice fed a high-iron diet for 8 days. As a control, no immuno-detectable band was observed in Lf immunoprecipitated from brains of APP–/– mice. The data indicate that holo-Lf is found in the normal brain and is augmented by dietary iron.

### Holo-Lf binding to APP decreases cell-surface APP and induces internalisation of Lf

**Fig. 2:**
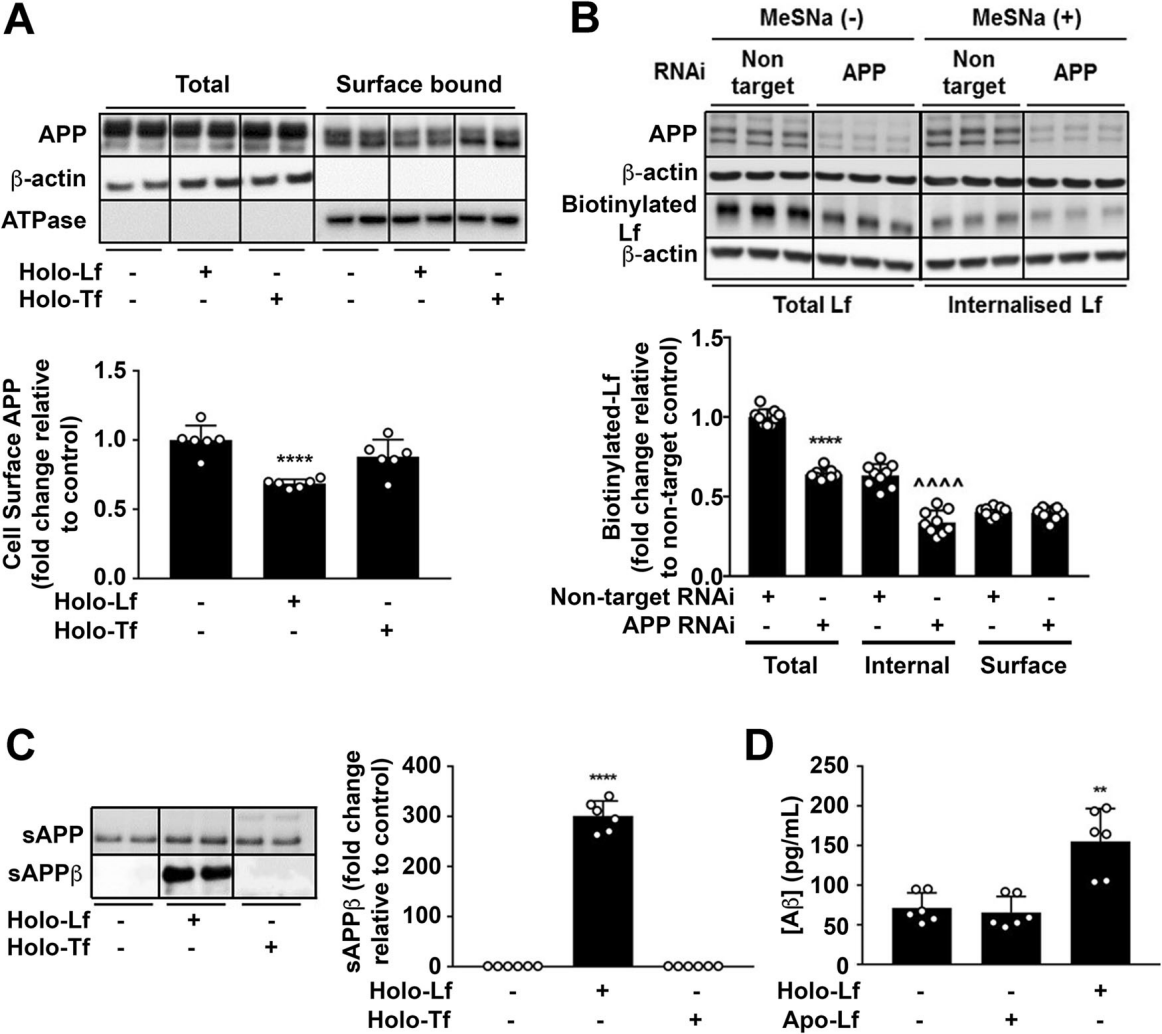
Iron-bound Lf decreases cell-surface APP levels and promotes the amyloidogenic processing of APP. **A** Biotinylation of surface proteins on primary murine neurons cultured in holo-Lf or holo-Tf (500 nM; 2 h) and followed by streptavidin immunoprecipitation shows a decrease in biotinylated APP with only holo-Lf when normalised against Na^+^/K^+^ ATPase surface protein content. **B** Biotinylated holo-Lf (200 nM; 1 h at 37 °C) was added to SH-SY5Y cells transfected with control non-target or APP RNAi (20 nM for 48 h) before being subjected to the ligand internalisation assay. APP depletion was confirmed by western blot (22C11). Total biotinylated holo-Lf (MeSNa (–)) and internalised holo-Lf (MeSNa (+)) (detected by Streptavidin-HRP) were quantified whilst surface-bound biotinylated Lf was determined by subtracting the internal from total fraction. **C** Primary murine neurons treated with holo-Lf or holo-Tf (500 nM; 2 h) were evaluated for sAPPβ release into the media. **D** Aβ production was also measured by ELISA on the media after treatment with apo- or holo-Lf (500 nM; 2 h). Data are mean ± SEM of three separate experiments performed in duplicate (**A**, **B**, **D**) or triplicate (**C**). Quantified data depict fold change compared to non-treated control cells, **p < 0.01 and ****p < 0.0001 or in (**B**) the non-targeted internal fraction, ^^^^*p* < 0.0001, as analysed statistically by two-tailed *t*-tests.

Given the association between APP and Lf, we examined the impact of holo-Lf applied extracellularly on the behaviour of APP in neuronal cell culture. Treatment with holo-Lf (500 nM; 2 h) selectively altered the location of APP in the neuron, decreasing surface APP (*p* < 0.0001) (Fig. 2A and Supplementary Fig. S3A). By contrast, neither holo-Tf nor the non-iron-bound forms of Lf or Tf impacted cell-surface APP as measured by biotinylation (Fig. 2A) or fluorescence-activated cell sorting (FACS) (Supplementary Fig. S3A).

To investigate the impact of APP on Lf endocytosis, a ligand internalisation assay was performed by adding biotinylated holo-Lf (200 nM; 1 h) to the media of SH-SY5Y cells with or without APP depletion (RNAi; 20 nM) (Fig. 2B). Once washed cells were stripped of cell-surface ligands by treatment with the cell-impermeable reducing agent (2-mercaptoethane sulfonic acid (MeSNa)), residual biotinylated Lf levels, reflecting internalised protein, were shown to be markedly decreased in APP depleted cells (*p* < 0.0001). Thus, cell-surface APP promotes holo-Lf internalisation.

### Holo-Lf promotes the amyloidogenic processing of APP

Amyloidogenic processing predominantly requires full-length membrane-bound APP to be internalised within an acidic endosomal vesicle for cleavage by β-secretase (BACE1) to generate soluble APPβ (sAPPβ). Subsequent cleavage by the γ-secretase complex produces Aβ that is secreted out of the cell [45].

The impact of holo-Lf binding to APP on amyloidogenic processing was examined in both mouse primary cultures and a human neuroblastoma line. In primary neurons, holo-Lf (500 nM; 2 h) strikingly increased sAPPβ (Fig. 2C) and Aβ (Fig. 2D) release into the media, and also increased intracellular Aβ levels (Supplementary Fig. S3C). No change in APP cleavage was observed when the cells were treated with either holo-Tf (Fig. 2C and Supplementary Fig. S3B) or apo-Lf (Fig. 2D and Supplementary Fig. S3C). The dramatic increase in amyloidogenic processing of APP induced by holo-Lf was dose- (Supplementary Fig. S4A) and time-dependent (Supplementary Fig. S4B) in the human neuroblastoma line.

### Holo-Lf-mediated APP internalisation is diverted to a clathrin-independent mechanism involving ARF6

**Fig. 3:**
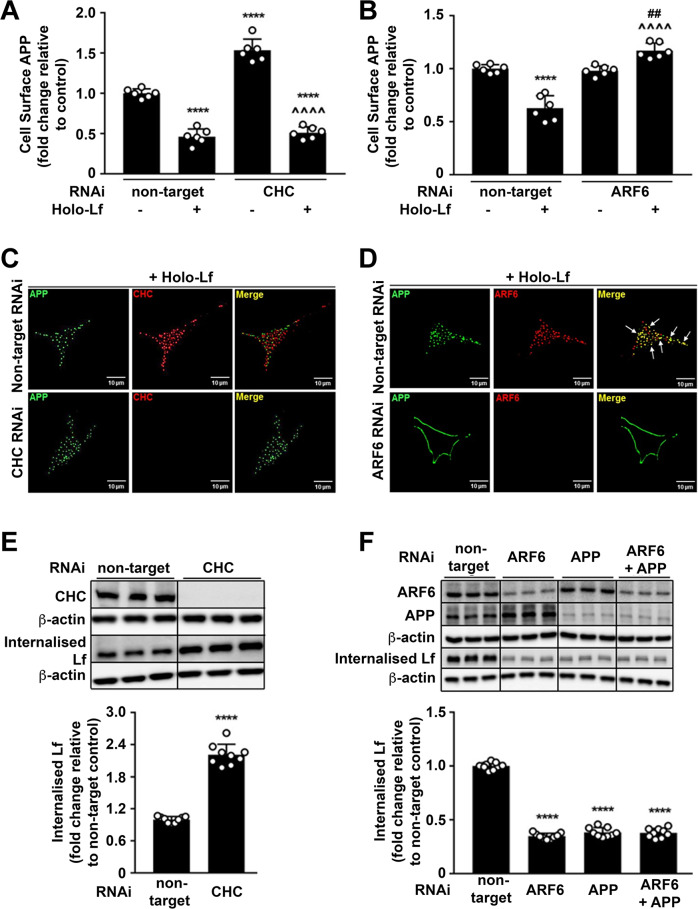
Holo-Lf-mediated APP internalisation is clathrin independent and ARF6 dependent. **A**, **B** Cell-surface APP (ab15272) response to holo-Lf (500 nM; 2 h) as monitored by FACS in non-permeabilised SH-SY5Ys after control non-targeted of CHC knockdown by RNAi (40 nM; 72 h) (**A**) or ARF6 RNAi (20 nM; 48 h) (**B**). **C**, **D** Deconvoluted images of double immunofluorescence confocal microscopy of wt-APP^695^ SH-SY5Ys treated with holo-Lf. After RNAi depletion of CHC or ARF6 as in (**A**, **B**), surface APP was labelled with an APP antibody (22C11) (green) at 4 °C before replacing media with holo-Lf (1 μM; 1 h) at 37 °C. An APP secondary detection antibody was then added with total CHC (ab21679) (red) (**C**) or ARF6 (ab131261) (red) (**D**) co-labelling. Co-localisation of APP with CHC (**C**) and ARF6 (**D**) is represented as yellow in the merged image (white arrows). **E**, **F** Biotinylated holo-Lf (0.5 mg/ml; 1 h at 37 °C) added to SH-SY5Ys transfected with control non-target and CHC (40 nM; 72 h) (**E**) or ARF6 ± APP RNAi (20 nM; 48 h) (**F**) before being subjected to the ligand internalisation assay. Residual surface biotin was stripped with MeSNa so that only internalised biotinylated Lf could be detected in the total cell lysate when analysed by western blot with streptavidin-HRP. Data are mean ± SEM of three experiments performed at least in duplicate. Statistical analysis in **A**, **B** was by two-way ANOVA or two-tailed *t*-tests for **E**, **F**, ****p < 0.0001 depicts fold change compared with levels derived from non-targeting control, ^^^^*p* < 0.0001 compared to CHC RNAi (**A**) or holo-Lf-treated (**B**) non-target control and ^##^*p* < 0.0001 compared to ARF6 RNAi (**B**). **C**, **D** Images are a representative from multiple cells within experiments carried out in duplicate. Scale bar = 10 µm.

APP internalisation is typically via clathrin-dependent endocytosis [46, 47] and inhibition of this pathway elevates APP levels on the cell surface [48], whereas BACE1 internalisation is via the endocytic trafficking regulator ARF6 [49, 50]. Indeed, impairment of clathrin-dependent endocytosis in the absence of holo-Lf by depletion of clathrin heavy chain (CHC RNAi; 40 nM) (Fig. 3A and Supplementary Fig. S5A, D) or dynamin (DYM RNAi; 20 nM) (Supplementary Fig. S5C) elevated the expression of APP on the cell surface, but ARF6 depletion (RNAi; 20 nM) (Fig. 3B and Supplementary Fig. S5B, E) had little effect. In contrast, in the presence of holo-Lf depletion of either CHC or DYM had little additional effect on the cell-surface APP levels, which were already suppressed by holo-Lf alone (Fig. 3A and Supplementary Fig. S5A, C). However, ARF6 depletion negated the loss in surface APP induced by holo-Lf (Fig. 3B and Supplementary Fig. S5B), consistent with holo-Lf inducing the internalisation of APP through the same endocytic pathway as BACE1.

Double immunofluorescence confocal microscopy was also used to monitor the internalisation of cell-surface APP induced by holo-Lf when either CHC- or ARF6-regulated endocytosis was inhibited. SH-SY5Y cells overexpressing wt-APP^695^ were pre-treated with RNAi for either CHC or ARF6, then surface-labelled with an N-terminal APP antibody (22C11) at 4 °C, washed and incubated with holo-Lf (1 μM; 1 h) at 37 °C. Immunofluorescent detection of APP, CHC or ARF6 was subsequently carried out on permeabilised cells. Internalised APP induced by holo-Lf did not colocalise with CHC and was not impaired by CHC depletion (Fig. 3C). In contrast, co-localisation of APP and ARF6 could be clearly observed in the presence of holo-Lf, while depletion of ARF6 strikingly induced APP to persist at the cell surface (Fig. 3D).

Consistent with APP acting as a surface-capture receptor for holo-Lf, the levels of internalised holo-Lf were markedly increased when cells were treated with CHC RNAi and subsequently challenged with biotinylated holo-Lf (Fig. 3E and Supplementary Fig. S5F). RNAi of ARF6 or APP impaired holo-Lf incorporation into the cell after 1 h and the lack of a combinatory effect upon double ARF6/APP knockdown was consistent with holo-Lf being internalised through the ARF6 machinery after being captured by APP (Fig. 3F).

### Holo-Lf promotes APP trafficking through the Rab11-positive recycling endosome

**Fig. 4:**
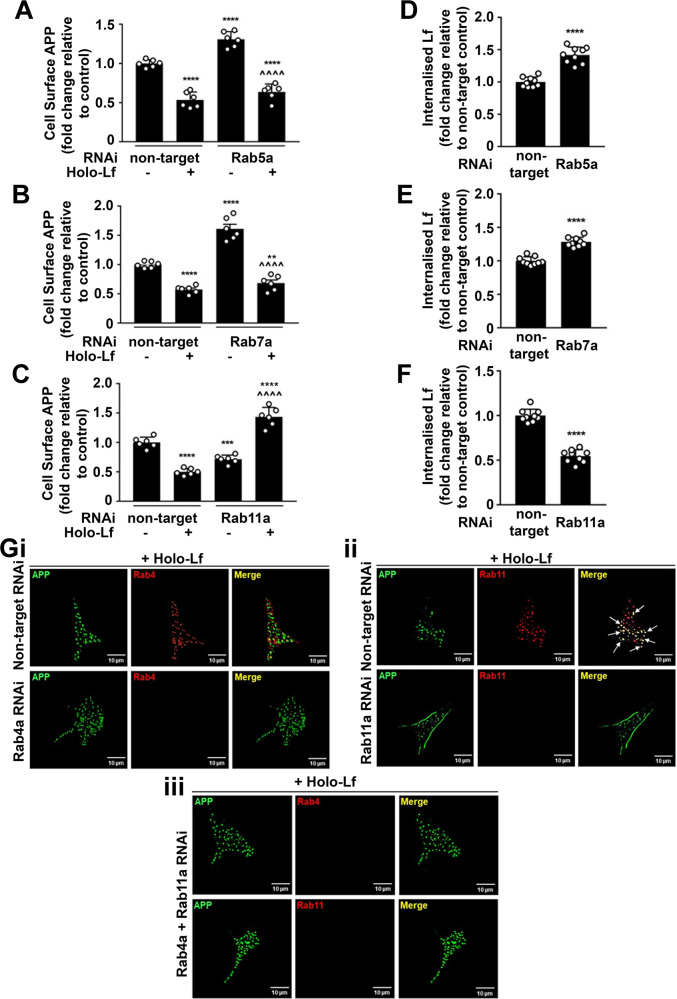
Holo-Lf promotes APP trafficking through the Rab11-positive recycling endosome. **A**–**C** Flow cytometric quantification of cell-surface APP levels (ab15272) on the cell surface of non-permeabilised SH-SY5Ys with and without holo-Lf (500 nM; 2 h) after treatment with RNAi (20 nM; 48 h) for Rab5a (**A**), Rab7a (**B**) Rab11a (**C**) and a non-targeted control. **D**–**F** Within the same experimental parameters as **A**–**C**, quantification of the effect of Rab5a (**D**), Rab7a (**E**) and Rab11a (**F**) knockdown on internalisation of biotinylated holo-Lf (0.5 mg/ml; 1 h at 37 °C) was measured by the ligand internalisation assay. Residual surface biotin was stripped with MeSNa so that only internalised biotinylated Lf could be detected in the total cell lysate when analysed by western blot (shown in Supplementary Fig. S6). **G** Representative deconvoluted images from double immunofluorescence confocal microscopy of wt-APP^695^ SH-SY5Ys reverse transfected with RNAi for control non-target, Rab4a (**i**) or Rab11a (**ii**). In double knockdown, cells were reverse transfected with Rab4a and then forward transfected with Rab11a (**iii**) RNAi (20 nM; 48 h). After surface labelling with anti-APP (22C11) (green) at 4 °C, cells were treated with holo-Lf (1 μM; 1 h at 37 °C) and then permeabilised to label with the antibody to APP (green) and anti-Rab4 (ab13252; red) (**i**, **iii**) or anti-Rab11 (ab3612; red) (**ii**, **iii**). **A**–**F** Data are mean ± SEM of three experiments performed at least in duplicate. Statistical analysis by two-way ANOVA (**A**–**C**) or two-tailed *t*-tests (**D**–**F**), **p < 0.01, ***p < 0.001 and ****p < 0.0001 depict fold change compared to the untreated non-targeting control and ^^^^*p* < 0.0001 compared to Rab RNAi without holo-Lf. **G** Images are a representative from multiple cells within experiments carried out in duplicate. Scale bar = 10 µm.

To further characterise the trafficking of this protein complex through the endosomal pathway, cell locations of APP and holo-Lf were analysed upon depletion of Rab5a (early endosome regulator [51]), Rab7a (a late endosome regulator [52]) and Rab11a (regulator for recycling endosomes [53]). Confirming prior studies (in the absence of holo-Lf), RNAi treatment (20 nM; 48 h) of Rab5a (Fig. 4A and Supplementary Fig. S6A) or Rab7a (Fig. 4A, B and Supplementary Fig. S6A, B) increased levels of APP on the cell surface, while depletion of Rab11a (Fig. 4C and Supplementary Fig. S6C) suppressed cell-surface APP. Upon holo-Lf treatment RNAi suppression of Rab5a or Rab7a had little effect on surface APP levels, whereas RNAi suppression of Rab11a markedly increased cell-surface APP levels (Fig. 4A, C and Supplementary Fig. S6A–C). In association with cell-surface APP levels, internalisation of biotinylated holo-Lf (200 nM; 1 h) was increased upon depletion of Rab5a (Fig. 4D) or Rab7a (Fig. 4E) but inhibited when Rab11a expression was depleted (Fig. 4F).

The changes observed with Rab11a depletion in the presence of holo-Lf (Fig. 4C, F) prompted further investigation of the endocytic recycling pathway. Rab4a (a regulator of ‘fast’ cargo recycling from the early/sorting endosome to plasma membrane [54]) was explored in conjunction with Rab11a using double immunofluorescence confocal microscopy. In wt-APP^695^-transfected SH-SY5Ys depleted of Rab4a, Rab11a or both (20 nM RNAi of each; 48 h) and then treated with holo-Lf (1 μM; 1 h at 37 °C), APP co-localised with Rab11a but not Rab4a-positive vesicles (Fig. 4Gi, ii). Furthermore, while Rab4a depletion alone had little effect on APP internalisation (Fig. 4Gi), the increased level of cell-surface APP upon Rab11a depletion (Fig. 4C, Gii) was negated when Rab4a was also knocked down (Fig. 4Giii). Taken together, these results indicate that holo-Lf when bound to APP is transported through an endocytic pathway independent of CHC, DYM, Rab5a or Rab7a but requiring ARF6 trafficking to the Rab11a-positive recycling endosome. However, when Rab11a is depleted, APP is diverted via Rab4a-positive endosomes to be rapidly transported back to the plasma membrane as a full-length protein.

### Holo-Lf accelerates secretion of amyloidogenic fragments of APP by diverting APP to the recycling endosome

**Fig. 5:**
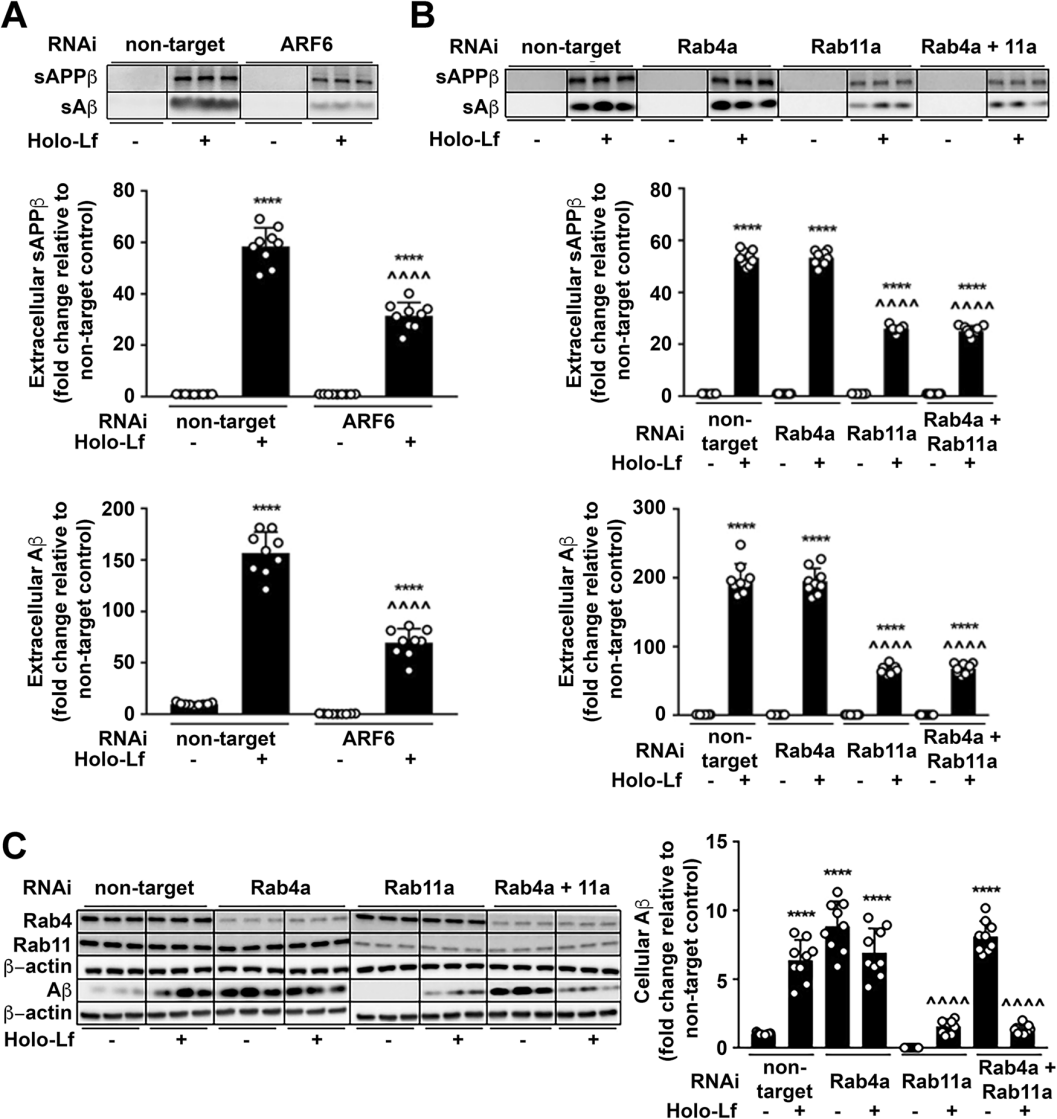
Holo-Lf-mediated amyloidogenic processing of APP requires ARF6 and the Rab11-positive recycling endosome. **A** Amyloidogenic processing of APP induced by holo-Lf (500 nM; 2 h) in wt-APP^695^ SH-SY5Ys pre-treated with control non-target or ARF6 RNAi (20 nM; 48 h) was measured by sAPPβ and Aβ levels in the media. **B**, **C** wt-APP^695^ SH-SY5Ys were reverse transfected with control non-target or Rab4a RNAi and/or forward transfected with Rab11a RNAi (20 nM; 48 h) before addition of holo-Lf (500 nM; 2 h). Extracellular sAPPβ and Aβ (**B**) as well as intracellular Aβ (**C**) protein levels were quantified. Data are mean ± SEM of three experiments performed in triplicate and depicted as fold change compared with levels derived from control non-target cells. Statistical analysis was by two-way ANOVA compared to untreated non-targeting control, ****p < 0.0001 or holo-Lf-treated non-targeting control, ^^^^*p* < 0.0001.

The consequence of holo-Lf-induced changes to APP trafficking through the endosomal pathway was evaluated by monitoring the APP amyloidogenic fragment profile. In the absence of holo-Lf, depletion of all the previously described modulators of endocytosis caused a decrease in sAPPβ and Aβ secretion into the media (ARF6; Fig. 5A, CHC; Supplementary Fig. S7B, DYM; Supplementary Fig. S7C, Rab5a; Supplementary Fig. S8A, Rab7a; Supplementary Fig. S8B, Rab4a and Rab11a; Fig. 7B). Intracellular Aβ was similarly decreased (ARF6; Supplementary Fig. S7A, CHC; Supplementary Fig. S7D, DYM; Supplementary Fig. S7E, Rab5a; Supplementary Fig. S8C, Rab7a; Supplementary Fig. S8D, Rab11a; Fig. 5C) except upon Rab4a knockdown (in the presence or absence of Rab11a), where Aβ retention was elevated (Fig. 5C).

The elevated production and excretion of both sAPPβ and Aβ induced by holo-Lf (500 nM; 2 h) (Fig. 2) was further increased when CHC, DYM, Rab5a or Rab7a were knocked down (Supplementary Figs S7B–E and S8). In contrast, ARF6 (Fig. 5A and Supplementary Fig. S7A) and Rab11a (Fig. 5B, C) depletion attenuated the sAPPβ and Aβ production and excretion response to holo-Lf but Rab4a depletion (Fig. 5B, C) did not affect the amyloidogenic processing pathway.

In combination with full-length APP and holo-Lf trafficking studies (Figs 3 and 4), these results show that a pathway involving CHC, DYM, Rab5a and Rab7a is not predominantly required for the amyloidogenic processing of APP in the presence of holo-Lf. An alternative pathway, likely involving ARF6 and Rab11a, has intracellular Aβ production induced by holo-Lf occurring within the Rab11a-positive recycling endosome. While Rab4a may have an effect on recycling APP back to the cell surface when Rab11a-dependent endosomal trafficking is impaired, it does not have an effect on the amyloidogenic processing of APP or secretion of sAPPβ and Aβ.

### Activated microglia secrete Lf to promote amyloidogenic processing of neuronal APP non-autonomously

**Fig. 6:**
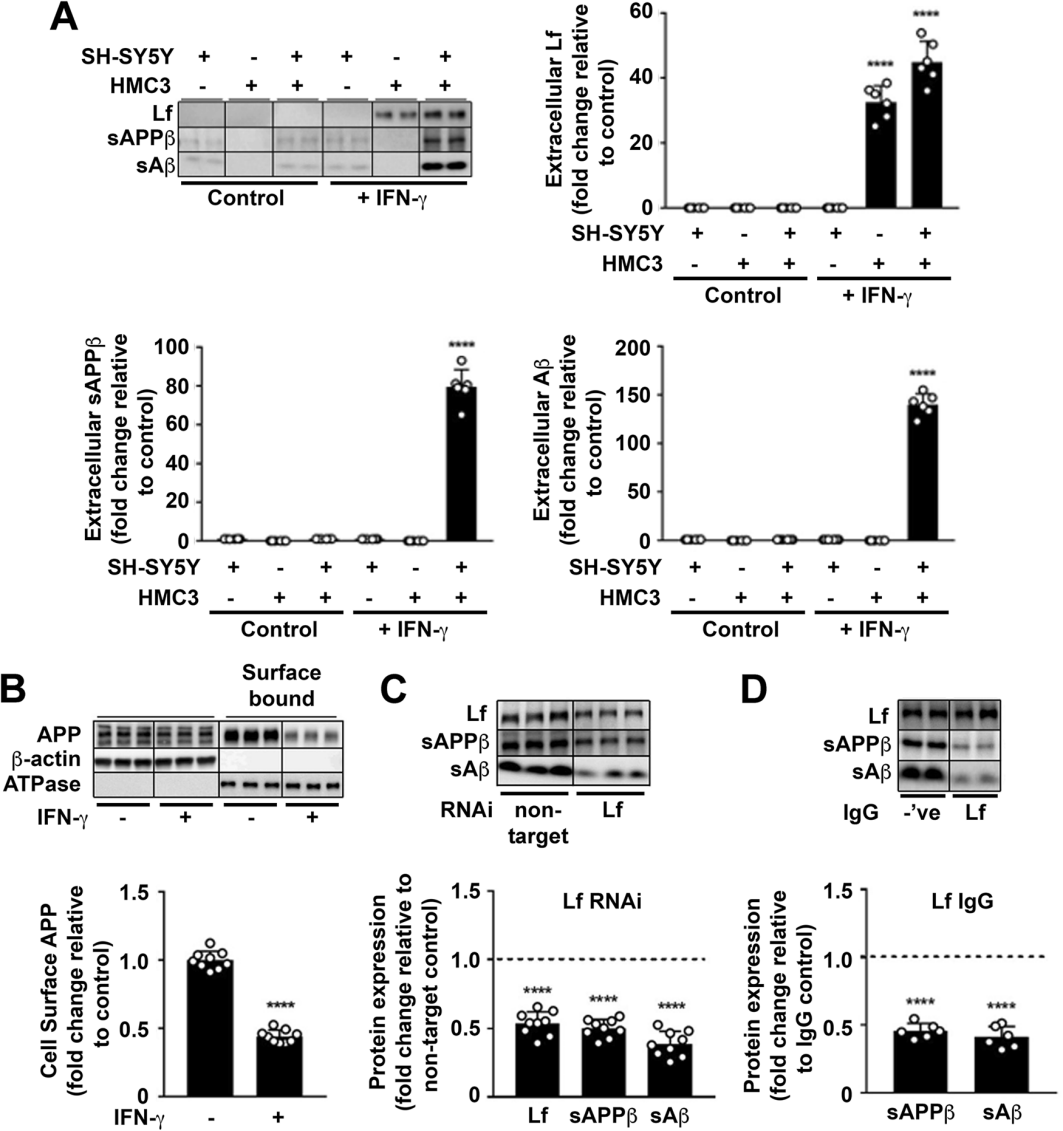
Secreted holo-Lf from activated microglia reduces neuronal surface-presented APP and increases APP amyloidogenic processing. **A** In monocultures and a transwell co-culture with HMC3 microglia cultured in the upper inserts and wt-APP^695^ SH-SY5Ys in the lower wells, human recombinant IFN-γ (10 ng/ml; 24 h) was used to activate microglia, as confirmed by the MHC class II marker (Supplementary Fig. S9A). IFN-γ-induced changes in media levels of secreted Lf, sAPPβ and Aβ were quantified from western blotting. **B** From transwell co-culture as in (**A**), surface APP from wt-APP^695^ SH-SY5Y co-cultures were measured by surface biotinylation after microglial activation. **C** HMC3 cells transfected with control non-target or Lf RNAi (20 nM; 48 h) were added to transwell co-cultures with wt-APP^695^ SH-SY5Y and activated with IFN-γ (10 ng/ml; 24 h) to determine expression levels of sAPPβ and Aβ secretion from wt-APP^695^ SH-SY5Y. **D** In a neutralising antibody inhibition assay, a polyclonal for Lf or an isotypic control IgG (20 μg/ml) was added to the media of wt-APP^695^ SH-SY5Y in the transwell co-culture before microglial activation by IFN-γ (10 ng/ml; 24 h). Inhibition of Lf binding to APP by the antibody was determined by measuring secreted Lf and the APP amyloidogenic protein fragments (sAPPβ and Aβ) in neuronal media. Data are mean ± SEM of three experiments performed in duplicate as a minimum and normalised against a control protein. Statistical analysis was by two-tailed *t*-tests compared to corresponding cell line without IFN-γ (**A**, **B**), non-target control (**C**) or isotype IgG treatment (**D**), ****p < 0.0001.

Brain Lf originates from peripheral Lf that crosses the blood–brain barrier by receptor mediated transport [55], but is also secreted from activated microglia [56] but not neurons. We, too, were unable to identify conditions in which Lf was expressed from neurons and so tested whether Lf secreted upon microglial activation was able to non-autonomously induce neuronal Aβ production. Using transwell co-cultures, stimulation by IFN-γ (10 ng/ml; 24 h) only induced Lf secretion when microglia (HMC3) were present. In turn, the amyloidogenic cleavage products of APP (sAPPβ, Aβ) were detected only when microglia were activated in the presence of neurons (wt-APP^695^ SH-SY5Ys) (Fig. 6A and Supplementary Fig. S9A). In the presence of microglia activated by IFN-γ, Aβ production was confirmed to originate from neurons since surface APP was decreased (Fig. 6B) and there was no detectable Aβ within the lysates of activated microglia after 24 h (Supplementary Fig. S9A).

To confirm that the neuronal APP amyloidogenesis was caused specifically by Lf and not some other secretory signal upon microglial activation, Lf immunoprecipitated from the media of activated microglia (Supplementary Fig. S9Bi) was added to wt-APP^695^ SH-SY5Y monocultures (Supplementary Fig. S9Bii). Neurons treated with immunoprecipitated Lf (2 h) expressed a significant increase in sAPPβ (Supplementary Fig. S9Biii) and Aβ secretion (Supplementary Fig. S9Biv), compared to the immuno-depleted media from activated microglia or an alternative IgG used as an immunoprecipitation negative control. Furthermore, Lf depletion by prior RNAi treatment (20 nM; 48 h) suppressed the ability of activated microglia (IFN-γ 10 ng/ml; 24 h) to induce co-cultured neuronal sAPPβ and sAβ secretion (Fig. 6C).

To directly investigate whether the downstream consequence of APP amyloidogenic cleavage was due to the binding of microglial Lf to APP on the neuronal cell surface a neutralising polyclonal Lf antibody (20 μg/ml; 24 h) was introduced into the transwell co-culture media. Upon activation of microglia by IFN-γ (10 ng/ml, 24 h), the presence of this neutralising antibody had no effect on secreted Lf levels but decreased the levels of sAPPβ and Aβ in the media compared to an isotypic IgG control (Fig. 6D). Together, these data reveal that Lf secreted from IFN-γ-activated microglia induce amyloidogenic APP processing by binding to neuronal cell-surface APP. Impeding Lf expression or disrupting Lf binding to APP attenuates this.

### Mapping the APP binding sites for holo-Lf critical for amyloidogenic processing

**Fig. 7:**
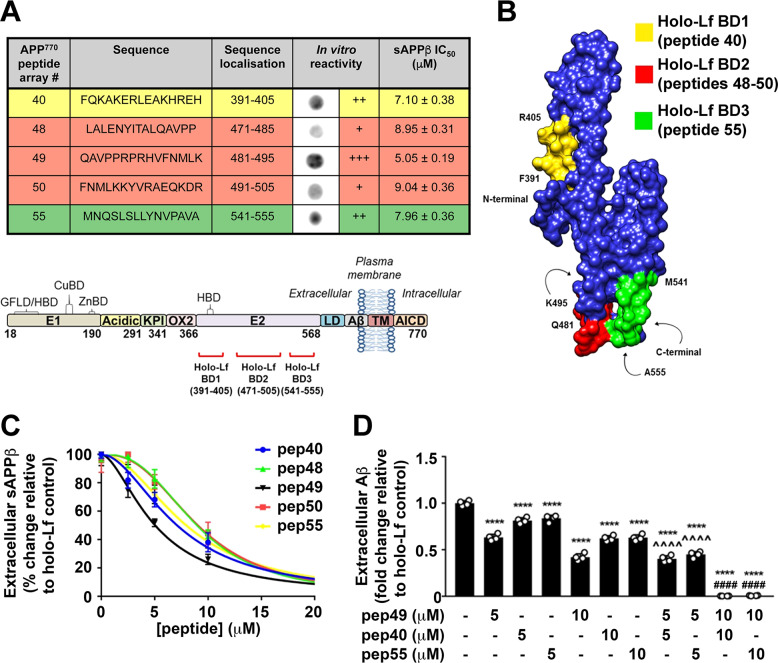
Identification of the holo-Lf binding sites on APP required for holo-Lf-induced amyloidogenic processing of APP. **A** APP peptide reactivity to holo-Lf (1 μg/ml; 2 h) was determined by visual observation (+++ strong, ++ moderate, + weak) and specificity of binding to holo-Lf by peptides 40, 48, 49, 50 and 55 was confirmed using apo-Lf and detection antibody treatment alone (see Supplementary Fig. S10A). **B** Model of holo-Lf binding sites (coloured as shown in (**A**)) overlaid on the APP-E2 structure [97]. **C** Potency of the holo-Lf binding peptides of APP was assessed by dose dependently pre-incubating the peptide with holo-Lf (500 nM; 2 h) in vitro before adding to wt-APP^695^ SH-SY5Y and evaluating sAPPβ secretion in the media after a further 2 h. wt-APP^695^ SH-SY5Y exposure of APP peptide alone at each respective concentration showed no change in sAPPβ levels (data not shown). IC_50_ for each peptide is shown in (**A**). **D** Covering the main holo-Lf binding sites, APP peptide 40, 49 and 55 were used to evaluate combinatory inhibition of holo-Lf-induced Aβ production in wt-APP^695^ SH-SY5Y. As in (**C**), peptide 49 with 40 or 55 (5 and 10 µM) was pre-incubated with holo-Lf (500 nM; 2 h) before addition to neuronal media for a further 2 h. Data are mean ± SEM of two experiments performed in duplicate with statistical analysis by two-way ANOVA comparing holo-Lf treated control, ****p < 0.0001, 5 µM peptide 49 alone, ^^^^*p* < 0.0001 or 10 µM peptide 49 alone, ^####^*p* < 0.0001.

To locate the minimal binding sites for holo-Lf on APP, purified human holo-Lf (1 μg/ml; 2 h) was incubated with 15-mer peptides from an array covering the full-length human wt-APP^770^ sequence (Supplementary Table S8) and attachment was assayed by immunodetection. Non-specific binding of Lf and IgG to peptides was controlled by incubating the array with apo-Lf (1 μg/ml; 2 h) and/or the primary and secondary antibodies (Supplementary Fig. S10A). Five peptides found to selectively bind holo-Lf were all located within the E2 domain of APP and corresponded to regions 391-405 APP^770^ (peptide 40), 471-505 APP^770^ (peptides 48-50) and 541-555 APP^770^ (peptide 55) (Fig. 7A, B).

To confirm the capability of these peptides to block amyloidogenic APP fragment production in a cell model, each peptide was dose dependently added to holo-Lf (500 nM; 2 h) before introduction into the media of wt-APP^695^ SH-SY5Ys for a further 2 h. The dose-dependent response to sAPPβ production (Fig. 7C and Supplementary Fig. S10B) correlated with the in vitro reactivity observed on the peptide array (Fig. 7A).

Amino acids 471-505 on APP^770^, in particular the region covered by peptide 49 (481-495 APP^770^), served as the major binding site for holo-Lf and was therefore used for combinatorial studies with peptides 40 (391-405 APP^770^) and 55 (541-555 APP^770^). sAPPβ and Aβ production in wt-APP^695^ SH-SY5Ys following the incubation of these peptides indicated that the presence of peptide 49 amplified inhibition by peptides 40 and 55 (i.e., 10 μM of peptides 40 or 55 vs. 5 μM of peptide 49 with 5 μM of peptide 40/55) but peptides 40 or 55 had little effect on peptide 49 inhibition (i.e., 10 μM of peptides 49 vs. 5 μM of peptide 49 with 5 μM of peptide 40/55) (Fig. 7D and Supplementary Fig. S10C). Observations indicate that of the two putative binding sites on full-length sAPP (Fig. 1E, F, Supplementary Fig. S2E–G and Supplementary Table S7), the major binding site for holo-Lf to induce neuronal amyloidogenic APP fragment production is within the region covered by peptide 49 (481-495 APP^770^) while the second, weaker, binding site on APP is likely to be within amino acids 391-405 or 541-555 of APP^770^.

## Discussion

Multivariate non-parametric analysis of gene expression data implicated several novel targets in classifying AD from controls, the most powerful of which was *LTF*, suggestively due to its strong association with Aβ pathology. Conventional DEA ranked *LTF* lowly (2360th), explaining why it was not featured in previous conventional analyses on the ROSMAP cohort [26–28]. The low ranking in the DEA appears to have been caused by abnormally high expression within just four of the controls and a vulnerability to such outliers has previously been highlighted when using parametric methods such as DEA [21]. In contrast, the non-parametric random forest algorithm used by Boruta is less susceptible to outliers and better suited for identifying genes such as *LTF* that show more consistent differences between groups [24]. While *LTF* was a promising hit through machine learning and consistently upregulated in AD, pathway databases (e.g., STRING) offered little insight into pathogenetic mechanism. So, we explored the impact of Lf on the well-understood biochemistry of Aβ generation from its precursor, APP.

Holo-Lf, but not apo-Lf, was discovered to bind to a motif within the E2 domain of APP (481-495 APP^770^), an extracellular region that has previously been identified as a heparin binding site [57]. Internalisation of the protein complex through an endocytic pathway to promote amyloidogenic processing of APP was mediated by ARF6 and Rab11a-positive endosomes. While this recycling vesicle has previously been reported to regulate APP amyloidogenic processing and is an interacting component in network analysis of GWAS on sporadic AD [58], this is in contrast to the primary route of Aβ production reportedly involving Rab5- [59] and Rab7-dependent [52] vesicles. One potential benefit for the neuron using this recycling compartment in amyloidogenic processing of APP is to allow Aβ to be rapidly generated and secreted to a location of functional relevance outside the cell. Antimicrobial properties of Aβ and its potential role in innate immunity are attracting increasing attention [6–8]. We propose that Aβ generation in response to holo-Lf could represent a protective response against real or incorrectly perceived infection [8, 16]. A similar mechanism in which amyloidogenic processing of APP is modulated by neuroinflammation requires interferon-induced transmembrane 3 to potentiate γ-secretase activity [60]. Lf elevation in response to a microbial insult may offer a mechanistic connection between an innate immune response and Aβ production as well as explain some of the acute protective mechanisms reported for Lf in AD [61, 62]. However, sustained production of Aβ under chronic Lf produced by the AD-related microgliosis (also potentially receiving contributions from astrocytosis or peripheral plasma) [31] may lead to Aβ deposition.

Microglia are the resident immune cells of the brain that become activated in response to infectious insult and other injuries [11, 16, 63]. The MHC class II marker of activated microglia in AD [64, 65] was induced by IFN-γ in our studies and led to the subsequent upregulation of Lf production and secretion by microglia, as previously described [56]. Lf is typically expressed at low levels in the brain by microglia but is rapidly transported across the blood–brain barrier rendering the demarcation between peripheral and endogenous expression of Lf in the brain difficult. However, it is known that levels of Lf in the brain increase with age [31] and upon activation of microglia [56]. While apo-Lf is thought to be the predominant form secreted from these cells, the residual levels of labile iron within the interstitial fluid of the brain could allow for holo-Lf formation and a non-autonomous response in neurons to rapidly induce Aβ production. In aged wild-type mice, as well as an APP transgenic mouse model, a high-iron diet induces Aβ production [66] and we show that a similar diet increases the binding of Lf to APP. Part of the innate immune role of Lf during a pathogen invasion is to deplete extracellular iron essential for pathogen replication [35] and therefore the burden of brain iron that drives the onset of sporadic AD [67–69] may particularly receive contributions from activated microglia [70–72], and is likely also to increase the proportion of iron-loaded holo-Lf within the interstitial space. Subsequently, the elevated holo-Lf would promote amyloidogenic processing of APP and thus contribute to amyloid pathology. One intriguing hypothesis we are considering relates to the recent discovery that Aβ is an antimicrobial peptide that may be purposefully produced in the acute phase response (e.g., [7]). The antimicrobial effect potentially might relate to the ability of Aβ to coordinate Fe^2+/3+^ to produce hydrogen peroxide and to foster toxicity [73]. Holo-Lf might not only force iron retention in neurons and promote amyloidogenic processing of Aβ, but the elevated iron levels might also bind to secreted Aβ in order to augment its microbiocidal activity. Further study will test this concept.

A better understanding of the physiological mechanism that induces the amyloidogenic processing of APP will help decipher the natural history of AD. Whether the elevated production of Aβ initially triggered in response to microglial secretion of Lf can lead to an amplification of the IFN-γ-relevant phenotype [60, 74] remains to be determined. In addition, other proinflammatory mediators secreted via an activated microglial phenotype [75, 76] might also provoke Aβ production. Pharmacological inhibition of the interaction between APP and Lf while still maintaining the other reported neuroprotective roles of Lf could provide a potential therapeutic for AD, but more work is needed to understand what the interaction between holo-Lf and APP represents.

## Materials and methods

### Biological reagents

Chemicals and reagents were all analytical grade or tissue culture grade and were obtained from Sigma-Aldrich, unless otherwise stated in Supplementary information.

### ROSMAP cohort description

This study was undertaken following permission from the Rush Alzheimer’s Disease Centre and data accessed via the Synapse website. Study data were provided by the Rush Alzheimer’s Disease Center, Rush University Medical Center, Chicago. Data collection was supported through funding by NIA grants P30AG10161, R01AG15819, R01AG17917, R01AG30146, R01AG36836, U01AG32984, U01AG46152, the Illinois Department of Public Health, and the Translational Genomics Research Institute. ROSMAP comprises two active longitudinal cohort studies that have recruited over 3300 individuals aged 65 and over with no known dementia in order to gather extensive clinical information prior to a comprehensive neuropathological examination at death. To date, 1475 cases had come to brain autopsy and quantitative Aβ as well as phosphorylated tau (AT8 positive) data from eight regions (hippocampus, entorhinal cortex, midfrontal cortex, inferior temporal, angular gyrus, calcarine cortex, anterior cingulate cortex and superior frontal cortex) combined with RNA-seq data from the dorsolateral prefrontal cortex was made available for 638 subjects. This study excluded cases with incomplete data or with documented presence of other forms of dementia. As 98.4% of the cases were from a non-Hispanic white background, the remaining 1.6% were also excluded in order to reduce a source of genetic variation. A total of 589 cases were included in the DEA, while 471 were included in the machine learning analysis due to missing regional pathology data.

The ROSMAP cohort participants were given a diagnoses of no AD, low likelihood of AD, intermediate likelihood of AD and high likelihood of AD according to modified NIA- Reagan criteria [77]. As in previous studies, cases with an intermediate or high likelihood of AD were deemed to meet the pathological criteria for AD (*n* = 354), while cases with low likelihood of AD or no AD pathology were designated as controls irrespective of a clinical diagnosis of dementia (*n* = 235) [28].

### Human tissue preparation and RNA-sequencing

As previously described, cDNA libraries were prepared using strand-specific dUTP and poly(A) selection from dorsolateral prefrontal cortex grey matter. They were sequenced on the Illumina HiSeq with 101-bp paired end reads and a mean coverage of 95 million reads [28]. Input fastq files for the present study were downloaded from https://www.synapse.org/#!Synapse:syn8612097. These fastq files had been reverted from binary alignment matrix files produced when the samples had been first aligned using Bowtie and included the mapped and unmapped reads from the original alignment. The fastq files were re-processed using our custom pipeline as follows: quality of the input data was assessed using Fastqc (version 0.11.3) before the reads were mapped to the GRCh38.p10 reference genome using the STAR (version 2.5.2a) aligner, potential transcripts identified using StringTie (version 1.3.3b) and known GENCODE genes quantified using RSEM (version 1.3.0) [78]. All analysis code is available at https://github.com/binfnstats/ROSMAP_RNAseq.

### Differential expression analysis

RSEM count data were imported into the R project environment. Reads with zero counts were filtered out, leaving 23,056 genes for downstream analyses. Outlier samples were identified using principal components analysis and hierarchical clustering and reads normalised using trimmed mean of means. An ANOVA was performed to find potential covariates with RNA integrity number, batch, age, gender and study (ROS or MAP) included in the differential expression model. DEA was then performed using EdgeR [78]. The EdgeR results were validated by running a second DEA with the same covariates using DESeq2 [79].

### Machine learning analysis

Reads were filtered and normalised using the same methods as for DEA and within the R project environment. A principal components analysis showed a clear batch effect (data not shown) and therefore counts were log-transformed prior to the removal of the batch effect using the ComBat algorithm [80]. These batch-corrected counts were then used as the input for Boruta and four machine learning algorithms (‘rpart’, ‘randomforest’, ‘xgbtree’ and ‘glmnet’) run using the ‘caret’ package [81]. The algorithms using ‘caret’ were run with five-fold cross-validation repeated 20 times. The Boruta algorithm is a wrapper built around the random forest machine learning algorithm used to identify all relevant features in a dataset [82]. Here, Boruta was run with maxruns set to 10,000 for both classification and regression. The classification run was performed to identify features of relevance to AD diagnosis and the regression runs were performed to identify the features relevant to total and regional amyloid. Pathway enrichment analysis was then carried out using the STRING platform [83] or WebGestalt with inputs of >2000 genes [84]. DEGs of interest were also compared against the Open Targets platform [85] (accessed 14 August 2019)

The upregulation of *LTF* in AD was validated using the AD Consensus Transcriptomics platform (https://swaruplab.bio.uci.edu/consensusAD). This is a searchable database used to compare the expression of genes of interest in AD, asymptomatic AD (subjects that meet the pathological criteria for AD but without having cognitive impairment) and controls using the Wilcoxon ranked sum test. The database includes ROSMAP (prefrontal cortex data that were also used in our analysis), MSSM (frontal pole, inferior frontal gyrus, parahippocampal gyrus and superior temporal gyrus) [86] and Mayo Clinic studies (temporal cortex) [87] along with three validation data sets (SFG and postcentral gyrus [42] and prefrontal cortex [88]).

### Recombinant protein production and purification

The recombinant fragments of the human soluble APP^695^α, APP^751^α and APP^770^α were all expressed in the methylotrophic yeast *Pichia pastoris* strain GS115 and purified as previously described [89]. Media containing APP required a two-step purification procedure using an AKTA FPLC (GE Healthcare), involving anion exchange on a Q-Sepharose column (1.6 × 25 cm column, GE Healthcare) followed by hydrophobic exchange with phenyl-Sepharose (0.5 × 5 cm column, GE Healthcare) [89]. Lf was purified from human skimmed breast milk following the procedure of Blackberg et al. [90]. Lf prepared by this method is predominantly in the apo form; however, to fully eliminate trace iron, samples were also incubated with sodium ascorbate before chromatographic purification. Saturation of iron in Lf to produce the holo-form was carried out with freshly prepared FeNTA solution (9.9 mM ferric nitrate, 8.5 mM nitrilotriacetic acid adjusted to pH 7.0) as previously described [91]. Unless otherwise stated, human Lf was predominantly used for experimental procedures.

### Sedimentation velocity

Sedimentation experiments were conducted using an XL-I analytical ultracentrifuge (Beckman Coulter Instruments) with an An-Ti60 rotor and double-sector 12-mm path length cells containing Quartz windows with charcoal-filled epon centrepieces. Samples were centrifuged at 50,000 rpm, with radial 280 nm absorbance scans acquired from 6 to 7.25 cm every 7 min. Data were analysed with the c(S) model from Sedfit 9.4 with maximum entropy regularisation to produce sedimentation coefficient distributions [92]. The resulting c(S) distributions were integrated to obtain the weight average sedimentation coefficient and dissociation constants were obtained from this data as previously described [93] (see Supplementary data).

### Tryptophan fluorescence

Tryptophan fluorescence spectra were measured in a 96-well quartz microplate using a Flexstation 3 fluorescence plate reader (Molecular Devices). Fluorescence spectra were acquired, in triplicate, using an excitation wavelength of 295 nm and scanning the emission monochromator from 320 to 450 nm in 1 nm steps, with a bandwidth of 5 nm and an averaging time of 1 s. Titration data of the change in fluorescence intensity at 340 nm as a function of Lf concentration were fitted to a model describing the binding of a ligand to two independent sites on an acceptor. As described by Bailey et al. [94] (see Supplementary data), the obtained estimates of the Kd_1:1_ and Kd_1:2_, were with the assumption that the change in fluorescence intensity is entirely representative of the fraction of complex formed.

### APP peptide array

Overlapping 15-mer peptides (the N-terminal five amino acids overlapped with previous peptide) covering the full length of APP^770^ were custom synthesised and spotted onto a cellulose membrane (GenScript). Before use, the peptide membrane was rehydrated in methanol (5 min at 37 °C) and thoroughly washed in PBS. The membrane was then blocked in PBS containing 0.1% (v/v) Tween-20 (PBS-T) with 3% (w/v) BSA (2 h at 37 °C) before holo-Lf was added to the blocking buffer (1 μg/ml, 24 h at 4 °C). Detection of bound Lf was by sequential incubation with rabbit α-Lf (2 h at 37 °C) and HRP-conjugated α-rabbit (1 h at 37 °C); both diluted in blocking buffer. The membrane was extensively washed in PBS-T between each step and HRP visualised with enhanced chemiluminescence (ECL) reagent using a LAS-3000 imaging suite to capture the images. To determine non-specific binding of holo-Lf, the above was repeated with recombinant apo-Lf as a substitute for holo-Lf, rabbit α-Lf alone or HRP-conjugated α-rabbit alone.

### Cell lines

Mouse primary neuronal cultures were prepared from isolated C57Bl/6 E14 embryos as per our previous report [5]. Human SH-SY5Y expressing endogenous APP or stably overexpressing APP^695^ (wt-APP^695^) were kindly provided by Prof. Anthony Turner (The University of Leeds, UK). Both neuroblastoma lines were maintained in complete growth medium consisting of Dulbecco’s Modified Eagle Medium containing 4.5 g/l glucose with L-glutamine (DMEM; Lonza) supplemented with 10% foetal bovine serum (FBS; Biosera). Human microglial clone 3 (HMC3) cells were purchased from American Type Culture Collection (ATCC^®^; CRL-3304™) and maintained in Eagle’s Minimum Essential Medium (EMEM; ATCC^®^) supplemented with 10% FBS. All cell lines were sustained in a humidified chamber at 37 °C with 5% CO_2_.

### Co-culture of wt-APP^695^ SH-SY5Y with microglial HMC3 cells

Transwell inserts from a 6-well plate (0.4 μm pore size; transwell plate; Corning Inc.) were pre-incubated with HMC3 complete growth medium at 37 °C overnight to improve cell attachment and distribution. wt-APP^695^ SH-SY5Y (8 × 10^5^ cells/ml) were plated onto well bottoms, while HMC3 (4 × 10^5^ cells/ml) were added to transwell inserts and pre-incubated overnight (37 °C, 5% CO_2_) in their respective complete growth media to allow cells to adhere. Inserts containing HMC3s were then placed into wells containing wt-APP^695^ SH-SY5Y to begin non-contact co-culture acclimatisation between cell types. For HMC3 activation, the medium of the upper insert well was replaced with serum-free EMEM containing recombinant human IFN-γ (10 ng/ml; PeproTech) for 24 h, while the medium in the lower plate well was replaced with fresh serum-free DMEM.

### RNA interference (RNAi)

In most experiments cells were seeded 1 day before being transiently forward transfected with RNAi (20 nM) directed against either APP, Lf, ARF6, DYM, Rab5a, Rab4a, Rab7a, Rab11a (SMARTpool: ON-TARGETplus Human siRNA; Horizon Discovery), or control non-target (ON-TARGETplus Non-targeting Pool; Horizon Discovery) using 4 μl of Lipofectamine^®^ RNAiMAX (Life Technologies) as described in the manufacturer’s instructions. In the case of HMC3, cells were re-plated into transwell inserts prior to activation by IFN-γ treatment and the inserts were subsequently placed into wells containing adhered wt-APP^695^ SH-SY5Ys.

Dual depletion of Rab4a and Rab11a required reverse transfection with Rab4a (20 nM; SMARTpool: ON-TARGETplus Human siRNA; Horizon Discovery) followed by forward transfection with Rab11a (20 nM; SMARTpool: ON-TARGETplus Human siRNA; Horizon Discovery) using 4 μL of Lipofectamine^®^ RNAiMAX for each treatment. Cells were incubated with the RNAi mixture for 48 h.

Lastly, a reverse transfection procedure was required for RNAi directed against CHC (40 nM; SMARTpool: ON-TARGETplus Human siRNA; Horizon Discovery) using 9 μL of Lipofectamine^®^ RNAiMAX (6 h at 37 °C). Media was replaced with complete growth medium and cells were incubated for a further 72 h.

### Preparation of cultured cell lysates and condensed media

Conditioned medium was collected and centrifuged (5 min; 2500 *g;* room temperature (RT)) to pellet unwanted cell debris. The supernatant was then concentrated approximately ten-fold using a 3 kDa molecular weight cut off Amicon Ultra-0.5 centrifugal filter unit with an Ultracel-3 membrane (Millipore) (14,000 *g* for 30 min at RT). Condensed media samples were stored at –20 °C until required.

Cells were washed with PBS and lysed in ice-cold RIPA buffer (150 mM NaCl, 1% (v/v) Nonidet P-40, 1% (w/v) sodium deoxycholate, 0.1% (v/v) SDS, 25 mM Tris-HCl, pH 7.6) with protease inhibitors (cOmplete^TM^, EDTA-free; Roche) for 15 min. Lysates were clarified by centrifugation (14,000 *g* for 15 min at 4 °C) and supernatants assayed for total protein content by BCA.

### Preparation of tissue lysates

Tissue extraction from mice was performed with the approval of the Institutional Animal Care and Use Committee and in accordance with statutory regulations. APP–/– mice [95] and background C57BL6/SV129 control male mice aged 12 months were used. For evaluation of samples from animals maintained on a high-iron diet, mice were administered freshly prepared carbonyl iron at 120 μg/g/day in an 8% sucrose solution for 8 days before sacrifice, whereas controls were administered with 8% sucrose solution only. Dose was orally applied with the use of an olive-tipped oroesophageal needle. PBS perfused brain tissue was homogenised in PBS containing 1% Triton X-100 (PBS-TX100) and centrifuged at 100,000 *g* for 30 min at 4 °C. Supernatants were measured for total protein content by BCA assay and total metal content by ICP-MS. In control wild-type mice iron levels were 21.2 ± 1.0 µg Fe/g protein and in iron fed mice levels were increased to 23.4 ± 1.5 µg Fe/g protein.

### SDS-PAGE and western blot

Ten microgram of total protein from cell media or lysate, or an equal volume of media when containing purified protein, was separated either on: 10% PAGE (Tris-Glycine; BioRad) for detection of sAPP/APP, sAPPβ, CHC, DYM and Lf, 4-20% PAGE (Tris-Glycine; BioRad) for detection of ARF6, Rab4, Rab5, Rab7, Rab11, MHC class II or 4–12% PAGE (Bis-Tris; Life Technologies) for Aβ detection. Protein transfer was typically to 0.45 μm polyvinylidene difluoride (PVDF) (Hybond-P; GE Healthcare Life Sciences) except for Aβ that was to 0.2 μm PVDF and Lf to 0.45 μm nitrocellulose (Protran; GE Healthcare Life Sciences). Membranes were boiled in PBS for 10 min to expose epitopes for antibody binding. Cooled membranes were probed with primary and secondary antibodies before visualisation by ECL reagent (Thermo Scientific) using a LAS-3000 imaging suite (Fujifilm Life Sciences). Densitometry using Image J was performed in triplicate on three separate experiments unless otherwise stated and all quantification was standardised against β-actin levels (1:5000) or Na^+^/K^+^ ATPase (1:5000) for surface protein analysis.

### Immunoprecipitation

For tissue, homogenised brain supernatant (100 μg) from each experimental condition was pre-cleared using protein G agarose beads (1 h at 4 °C). Pre-cleared supernatant was incubated with α-Lf (1:200; LifeSpan BioSciences) (1 h at 4 °C), before the addition of freshly equilibrated protein G agarose beads (2 h at 4 °C). Unbound supernatant was separated and stored before bound proteins were eluted from beads with SDS-PAGE loading buffer after thorough washing in PBS-TX100. Protein capture was visualised by western blotting.

Lf immunoprecipitation experiments on culture media was carried out using the Immunoprecipitation Dynabeads™ protein G kit (Thermo Scientific) as per the manufacturer’s instructions with minor modifications. After treatment to cells, the collected media was pre-cleared (1 h at 4 °C), incubated with capture antibody (α-Lf; 1:200; LifeSpan BioSciences) (1 h at 4 °C) before mixing with equilibrated protein G Dynabeads for a further 24 h at 4 °C. Unbound supernatant was saved for further analysis whilst bound proteins were eluted from beads with citrate buffer (100 mM; pH 2) (10 min at RT) after thorough washing in PBS-TX100. Eluted fractions were buffer exchanged with HBSS using Zeba™ Spin Desalting Columns and Lf levels confirmed by western blot using a rabbit anti-Lf (1:500; bs-5810R; Bioss) for detection before application to wt-APP^695^ SH-SY5Y.

### Cell-based protein interaction blocking studies

Selected 15-mer APP peptides from the peptide array were custom made with >95% purity (GenScript). Peptides were dissolved in DMSO and pre-incubated with holo-Lf in serum-free DMEM containing 0.01% BSA (2 h at 4 °C). Each peptide complex was then applied to the media of wt-APP^695^ SH-SY5Y for a further 2 h at 37 °C.

For antibody neutralisation experiments in co-culture studies, rabbit anti-Lf antibody or rabbit IgG isotype control (20 μg/ml; 24 h) was added with serum-free DMEM to wt-APP^695^ SH-SY5Y or with serum-free EMEM (±IFN-γ) to HMC3.

### Surface biotinylation assay

Cell-surface biotinylation was carried out as previously described [5] using the Pierce cell-surface protein isolation kit (Thermo Scientific) as per the manufacturer’s instructions. Briefly, cells were labelled with sulfosuccinimidyl-2-[biotinamido] ethyl-1,3-dithiopropionate (Sulfo-NHS-SS-Biotin) in PBS (Mg/Ca) for 30 min on ice, the unreacted biotin was quenched with 100 mM glycine and protein content in cells assayed by BCA after lysis. To precipitate biotinylated surface proteins, equal protein amounts from cell lysates were incubated with High Capacity Neutravidin Agarose (Thermo Scientific). Non-bound ‘intracellular’ proteins were separated before agarose was washed with ice-cold ‘high salt’ wash buffer (500 mM NaCl, 5 mM EDTA, 50 mM Tris-HCl pH 7.5) and ice-cold ‘no salt’ wash buffer (10 mM Tris-HCl pH 7.5). Bound proteins were eluted with SDS-PAGE sample buffer containing 50 mM DTT, before analysis by western blotting.

### Ligand internalisation assay

Human holo-Lf was labelled with 20-fold molar excess of Sulfo-NHS-SS-Biotin for 30 min at RT. Removal of non-reacted biotin was performed by gel filtration using Zeba™ Spin Desalting Columns with a 40 kDa molecular weight cut off (Thermo Scientific). Biotinylated holo-Lf (200 nM) was added to SH-SY5Ys (1 h at 37 °C) in serum-free media to allow internalisation and endocytosis was stopped with ice-cold NT buffer (150 mM NaCl, 1 mM EDTA, 0.2% BSA, 20 mM Tris-HCl pH 8.5) (5 min). Residual surface biotin was stripped with two washes (30 min each at 4 °C) of MeSNa (20 mM) in NT buffer. Cells were washed with ice-cold HBSS then incubated with HBSS containing 20 mM iodoacetic acid (15 min) to deactivate residual MeSNa. To examine total biotinylated Lf, the MeSNa treatment was omitted. Biotinylated holo-Lf was detected by western blotting using a Streptavidin-HRP antibody.

### Fluorescence-activated cell sorting (FACS)

To assess surface-presented APP levels on SH-SY5Ys, FACS was carried out as per our previous report [5] using ab15272 to determine cell-surface APP. Intensity of fluorescence was compared to cells stained with secondary alone to minimise the detection of non-specific binding. Cells were sorted by forward and side scatter on a BD-LSR-Fortessa (BD Biosciences) with a 488 nM blue laser according to fluorescence at 530 ± 30 nM. A minimum of 10,000 cells were recorded in each experiment, having gated the cell population to ensure that only live cells were monitored. Experiments were carried out in duplicate on three separate occasions and data were analysed using BD FACS DiVa 6.0 and FlowJo 7.6.4 software.

### Enzyme-linked immunosorbent assay (ELISA) for Aβ detection

Total Aβ levels in cell lysates and conditioned media were quantified using the double-antibody capture ELISA as previously described [96]. W02 was used as the capture antibody and HRP-conjugated 1E8 the detection antibody. Quantifiable Aβ values in each sample were calculated by comparison to a synthetic Aβ_1-42_ (Bachem) standard curve. Absorbance was read at 450 nm using a FlexStation 3 with SoftMax Pro 5.4.6 software (Molecular Devices).

### Double immunofluorescence and confocal microscopy

Treated wt-APP^695^ SH-SY5Ys on poly-L-lysine coated coverslips were blocked in DMEM containing 1% BSA (w/v) (30 min at 4 °C). Surface-presented APP was labelled by incubating the cells on ice with 22C11 (1:50) in blocking buffer for 20 min. The 4 °C step temporarily impairs APP internalisation to increase predominance of cell-surface APP labelling. These non-permeabilised cells were then rinsed in PBS and incubated with holo-Lf (1 μM) in HBSS (1 h at 37 °C). Cells were washed with PBS (Mg/Ca) and fixed with 4% paraformaldehyde (10 min at RT). Excessive fixative was quenched with 50 mM NH_4_Cl and cells permeabilised in PBS-TX100 (15 min at RT) to permit access to internalised proteins. Cells were blocked again in PBS (Mg/Ca) containing 5% donkey serum (v/v) (30 min at RT) before incubation with the appropriate primary antibodies diluted in blocking buffer to detect CHC (1:100), ARF6 (1:100), Rab4 (1:100) and Rab11 (1:50) (24 h at 4 °C). Cells were then incubated with Alexa Fluor 488 anti-Mouse IgG (Thermo Scientific) and Alexa Fluor 568 anti-Rabbit IgG (Thermo Scientific) (1:500; 1 h at 37 °C) followed by washes in PBS (Mg/Ca) and a ‘no salt’ wash buffer before counterstaining with DAPI (1∶2000; Cell Signaling Technology). Slides mounted using FluorSave™ mounting reagent (Millipore) were imaged using a Zeiss LSM700 inverted confocal microscope under an oil-immersed 63× objective lens (NA = 1.40). *Z*-stacks of immunofluorescent stained cells were deconvoluted using ZEN 2.3 SP1 (black) software (Carl Zeiss Microscopy GmbH) with each stack compiled using Image J software (v1.48k, NIH).

### Statistical analysis

Statistical analysis for all tissue culture experiments was performed with Microsoft Excel v15 and GraphPad Prism 8 software. Unless specifically stated, all multiple comparisons were analysed by two-way ANOVA with Bonferroni post-hoc analysis. Single comparisons between groups was carried out by a two-tailed *t* test.

## Supplementary information

Supplementary text

Supplementary Figure 1

Supplementary Figure 2

Supplementary Figure 3

Supplementary Figure 4

Supplementary Figure 5

Supplementary Figure 6

Supplementary Figure 7

Supplementary Figure 8

Supplementary Figure 9

Supplementary Figure 10

Supplementary Table 1

Supplementary Table 2

Supplementary Table 3

Supplementary Table 4

Supplementary Table 5

Supplementary Table 6
