# Supplementary material for: The acute phase protein lactoferrin is a key feature of Alzheimer’s disease and predictor of Aβ burden through induction of APP amyloidogenic processing
Source: Mol Psychiatry. 2021 Aug 16;26(10):5516–31. doi: 10.1038/s41380-021-01248-1 (PMC8758478; doi:10.1038/s41380-021-01248-1)
Supplement: Supplementary file 1 — Supplementary text [file 41380_2021_1248_MOESM1_ESM.pdf]

The acute phase protein lactoferrin is a key feature of Alzheimer's disease and predictor of Aβ burden through induction of APP amyloidogenic processing

Andrew Tsatsanis, Andrew N. McCorkindale, Bruce X. Wong, Ellis Patrick, Tim M. Ryan, Robert W. Evans, Ashley I. Bush, Greg T. Sutherland, Asipu Sivaprasadarao, Boris Guennewig and James A. Duce.

SUPPLEMENTAL INFORMATION

Table of Materials

REAGENT or RESOURCE	SOURCE	IDENTIFIER
Antibodies		
**Mouse monoclonal anti-Alzheimer Precursor Protein A4 (N-terminal), clone 22C11**	Millipore	Cat# MAB348
**Rabbit polyclonal anti-Amyloid Precursor Protein**	Abcam	Cat# ab15272
**Rabbit polyclonal anti-sAPPβ, clone Poly8134**	BioLegend	Cat# 813401
**Mouse monoclonal anti-β-Amyloid, clone 6E10**	BioLegend	Cat# 803001
**Rabbit polyclonal anti-Clathrin Heavy Chain**	Abcam	Cat# ab21679
**Rabbit monoclonal anti-Dynamin, clone EP801Y**	Abcam	Cat# ab52611
**Rabbit monoclonal anti-ADP Ribosylation Factor 6, clone EPR8357**	Abcam	Cat# ab131261
**Rabbit polyclonal anti-Rab4**	Abcam	Cat# ab13252
**Rabbit monoclonal anti-Rab5, clone EPR5438**	Abcam	Cat# ab109534
**Rabbit monoclonal anti-Rab7, clone EPR7589**	Abcam	Cat# ab137029
**Rabbit polyclonal anti-Rab11**	Abcam	Cat# ab3612
**Rabbit polyclonal anti-Lactoferrin**	Bioss	Cat# bs-5810R
**Rabbit polyclonal anti-Lactoferrin**	LifeSpan BioSciences	Cat# LS-B10980
**Rabbit monoclonal anti-Major Histocompatibility Complex class II antigen beta Chain, clone EPR11226**	Abcam	Cat# ab157210
**Mouse monoclonal anti-β-Actin, clone AC-15**	Sigma	Cat# A5441
**Mouse monoclonal anti-alpha 1 Sodium Potassium ATPase, clone 464.6**	Abcam	Cat# ab7671
**Pierce High Sensitivity Streptavidin-HRP**	Thermo Scientific	Cat# 21130
**Mouse monoclonal anti-Amyloid-β, clone WO2**	Millipore	Cat# MABN10

**Mouse monoclonal anti-Amyloid βA4 (Amino terminal), clone 1E8**	Millipore	Cat# MABN639
**Goat polyclonal anti-Rabbit IgG-HRP**	Sigma	Cat# A0545
**Rabbit polyclonal anti-Mouse IgG-HRP**	Sigma	Cat# A9044
**Goat polyclonal anti-Rabbit IgG, AlexaFluor 488**	Thermo Scientific	Cat# A-11008
**Donkey polyclonal anti-Mouse IgG, Alexa Fluor 488**	Thermo Scientific	Cat# A-21202
**Donkey polyclonal anti-Rabbit IgG, Alexa Fluor 568**	Thermo Scientific	Cat# A10042
Chemicals, Peptides and Recombinant Proteins		
**Recombinant human sAPP^{695}α**	[1, 2]	N/A
**Recombinant human sAPP^{751}α**	[1, 2]	N/A
**Recombinant human sAPP^{770}α**	[1, 2]	N/A
**Purified human Lactoferrin**	[3]	N/A
**Synthetic Amyloid β-Protein (1-42)**	Bachem	Cat# H-1368
**Iron(III) nitrate nonahydrate**	Sigma	Cat# F8508
**Nitrilotriacetic acid disodium salt**	Sigma	Cat# N0128
**APP^{770} Peptide Array**	GenScript	N/A
**Tween 20**	Sigma	Cat# P1379
**Bovine Serum Albumin**	Sigma	Cat# A2153
**Dulbecco's Modified Eagle Medium (DMEM)**	Lonza	Cat# BE12-604F
**Fetal Bovine Serum (FBS)**	Biosera	Cat# FB-1285
**Eagle's Minimum Essential Medium (EMEM)**	ATCC	Cat# 30-2003
**Recombinant Human IFN-γ**	PeproTech	Cat# 300-02
**Lipofectamine RNAiMAX**	Life Technologies	Cat# 13778030
**Sodium chloride**	Sigma	Cat# S9888
**Nonidet P-40**	Sigma	Cat# 56741
**Sodium deoxycholate**	Sigma	Cat# D6750
**Sodium dodecyl sulfate**	Sigma	Cat# L3771
**Tris**	Sigma	Cat# T6066
**cOmplete EDTA-free Protease Inhibitor Cocktail**	Roche	Cat# 4693159001
**Triton X-100**	Sigma	Cat# X100
**Paraformaldehyde, 16% w/v aq. soln.**	Alfa Aesar	Cat# 43368
**Ammonium chloride**	Sigma	Cat# A9434
**10% Mini-PROTEAN TGX Precast Gels**	BioRad	Cat# 4561036
**4-20% Mini-PROTEAN TGX Precast Gels**	BioRad	Cat# 4561096
**4-12% PAGE Bis-Tris Protein Gels**	Life Technologies	Cat# NP0336BOX

**Pierce ECL Western Blotting Substrate**	Thermo Scientific	Cat# 32209
**Rabbit IgG**	Sigma	Cat# I8140
**Ethylenediaminetetraacetic acid**	Sigma	Cat# E6758
**Sodium 2-mercaptoethanesulfonate**	Sigma	Cat# 63705
**Iodoacetic acid**	Sigma	Cat# I4386
**4',6-diamidino-2-phenylindol dihydrochloride (DAPI)**	Cell Signaling Technology	Cat# 4083
**Donkey serum**	Sigma	Cat# D9663
**FluorSave mounting reagent**	Millipore	Cat# 345789
Experimental Models: Cell Lines		
**Human SH-SY5Y**	ATCC	Cat# CRL-2266
**Human Microglial Clone 3**	ATCC	Cat# CRL-3304
RNA Interference (RNAi)		
**ON-TARGETplus Non-targeting Control Pool**	Horizon Discovery	Cat# D-001810-10-20
**SMARTpool: ON-TARGETplus Human Amyloid Precursor Protein**	Horizon Discovery	Cat# L-003731-00-0010
**SMARTpool: ON-TARGETplus Human Lactoferrin**	Horizon Discovery	Cat# L-019661-00-0010
**SMARTpool: ON-TARGETplus Human ADP Ribosylation Factor 6**	Horizon Discovery	Cat# L-004008-00-0010
**SMARTpool: ON-TARGETplus Human Dynamin**	Horizon Discovery	Cat# L-003940-00-0010
**SMARTpool: ON-TARGETplus Human Rab5a**	Horizon Discovery	Cat# L-004009-00-0010
**SMARTpool: ON-TARGETplus Human Rab4a**	Horizon Discovery	Cat# L-008539-00-0010
**SMARTpool: ON-TARGETplus Human Rab7a**	Horizon Discovery	Cat# L-010388-00-0010
**SMARTpool: ON-TARGETplus Human Rab11a**	Horizon Discovery	Cat# L-004726-00-0010
**SMARTpool: ON-TARGETplus Human Clathrin Heavy Chain**	Horizon Discovery	Cat# L-004001-01-0010
Critical Commercial Assays		
**Pierce BCA Protein Assay**	Thermo Scientific	Cat# 23225
**Immunoprecipitation Dynabeads Protein G Kit**	Thermo Scientific	Cat# 10007D
**Pierce Cell Surface Protein Isolation Kit**	Thermo Scientific	Cat# 89881

Supporting information on calculations required for dissociation constants

Sedimentation velocity analysis

In general, the sedimentation velocity data is fitted to the function:

$$Sw = \frac{1}{c_{tot}} \sum S_i c_i \qquad \text{Eq 1}$$

where Sw is the weight average sedimentation coefficient, c_{tot} is the total concentration of macromolecules, S_i is the sedimentation coefficient of component i, and c_i is the concentration of component i. The concentration of each component is related to the dissociation constant (Kd) of the complex by standard mass conservation equations [4].

Tryptophan fluorescence analysis

The fluorescence data was analysed using the following equation:

$$I_t = (fA \times IA) + (fL \times IL) + (fAL_1 \times IAL_1) + (fAL_2 \times IAL_2) + \left(fAL_{1,2} \times IAL_{1,2}\right) \qquad 1$$

where the total intensity (I_t) of the sample is given by the fraction (f) of APP alone (A), lactoferrin alone (L), APP and lactoferrin bound at site one (LA_1), APP with lactoferrin bound at site two (LA_2) and APP with lactoferrin bound at site one and site two ($LA_{1,2}$) multiplied by the intensity of each respective complex. The equilibrium concentration of each complex is determined by:

$$[A]_{free} = \frac{Kd_{1:1}Kd_{1:2}[A]_t}{Kd_{1:1}Kd_{1:2}+Kd_{1:1}[L]+Kd_{1:2}[L]+[L]^2} \qquad 2$$

$$AL_1 = \frac{[L][A]}{Kd_{1:1}} \qquad 3$$

$$AL_2 = \frac{[L][A]}{Kd_{1:2}} \qquad 4$$

$$AL_{1,2} = \frac{[L]^2[A]}{Kd_{1:1}Kd_{1:2}} \qquad 5$$

And

$$[L] = \frac{\alpha}{3} + \frac{2}{3}\sqrt{(\alpha^2 - 3b)}\frac{\theta}{3} \qquad 6$$

where

$$\alpha = Kd_{1:1} + Kd_{1:2} + 2[A]_t - [L]_t \qquad 7$$

$$b = Kd_{1:1}Kd_{1:2} + Kd_{1:1}[A]_t + Kd_{1:2}[A]_t - Kd_{1:1}[L]_t - Kd_{1:2}[L]_t \qquad 8$$

$$c = Kd_{1:1}Kd_{1:2}[L]_t \qquad 9$$

where $[A]_{free}$ is the free concentration of APP, $[A]_t$ is the total concentration of APP, [L] is the free concentration of lactoferrin, $[L]_t$ is the total concentration of lactoferrin, $Kd_{1:1}$ is the dissociation constant for binding to site one and $Kd_{1:2}$ is the dissociation constant for binding to site two. These equations are a general description of an independent two-site interaction. To apply these equations to the current model we assume that the change in fluorescence intensity is taken to be proportional to the fraction of each complex and that the binding of lactoferrin to site one is taken to provide the same fluorescence change as the binding of lactoferrin to site two.

SUPPLEMENTAL FIGURES

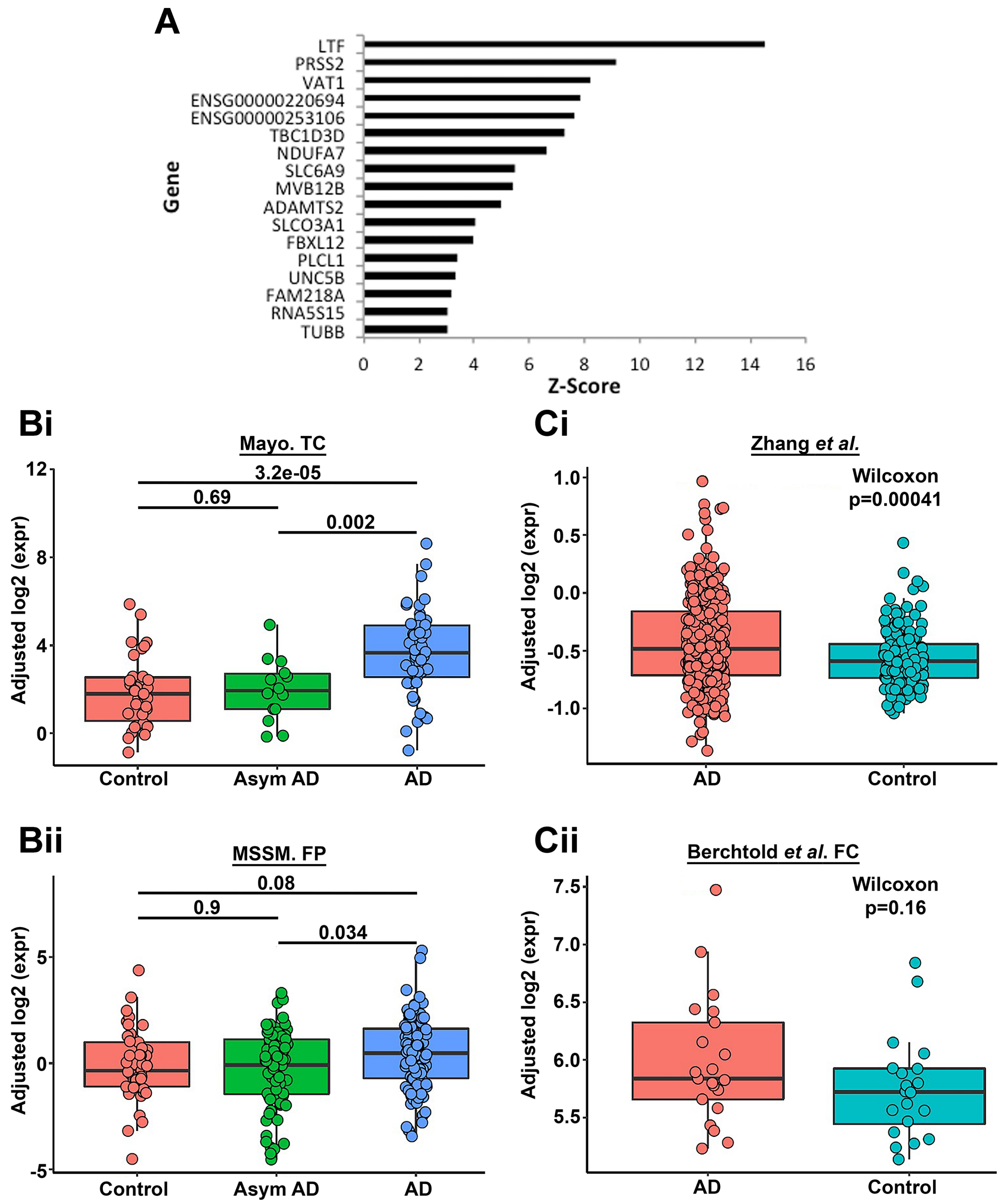

Figure S1. Random forest machine learning to predict Aβ pathology prediction in the ROSMAP cohort and expression of *LTF* in other publically accessible datasets. A. All genes ranked by z-score using the feature selection algorithm, Boruta as of relative importance for predicting amyloid load in the superior frontal gyrus**. B.** Expression of *LTF* in controls, asymptomatic AD and symptomatic AD in the Mount Sinai School of Medicine frontal pole (MSSM.FP)(**i**) and Mayo Clinic temporal cortex (Mayo.TC) (**ii**) datasets generated on the

AD consensus transcriptomics platform [5](https://swaruplab.bio.uci.edu/consensusAD). **C.** Expression of *LTF* in controls and AD in the Zhang *et al* [6] (**i**) and Berchtold *et al* [7] (**ii**) frontal cortex microarray datasets used for validation on the AD consensus transcriptomics platform (URL, subpage). The AD consensus transcriptomics platform compares expression between groups using the Wilcoxon ranked sum test **(B&C)**.

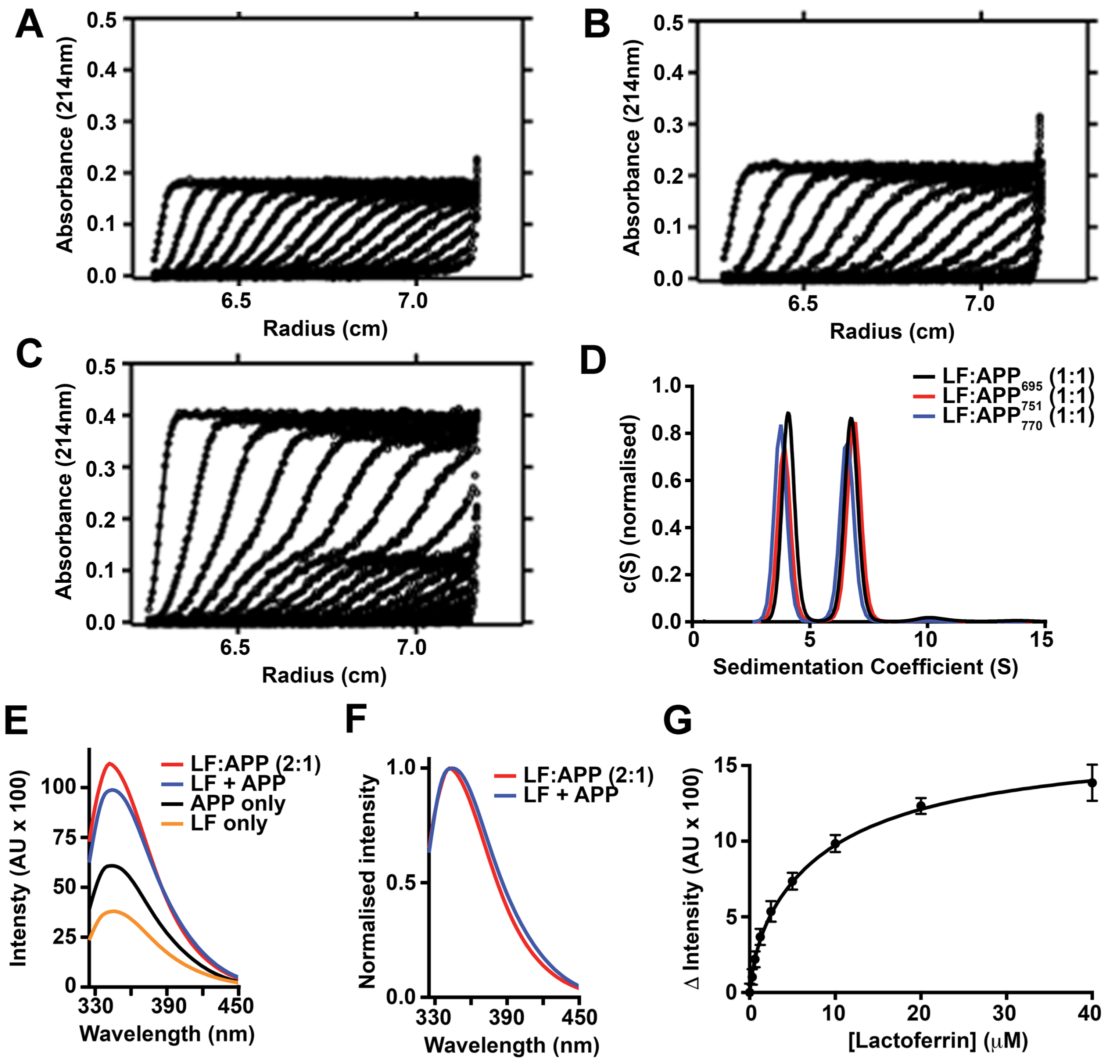

Figure S2. Lf directly interacts with APP. A-C. Representative sedimentation velocity raw data for APP alone **(A)**, Lf alone **(B)** and a mixture of APP and Lf **(C)** used to fit a c(S) model from sedfit 9.4 (solid lines through the data) to obtain the corresponding sedimentation coefficient distributions (**Fig.1E & S1D**). **D.** Sedimentation coefficient distributions of APP695 (black), APP751 (red) and APP770 (blue) (2.5 μM) in the presence of holo-Lf (2.5 μM). **E.** Tryptophan fluorescence spectra were acquired using an excitation wavelength of 295 nm for APP alone (2.5 μM) (black), holo-Lf alone (5 μM) (orange) and a mixture of APP and holo-Lf (2.5 μM and 5 μM, respectively) (red). To determine the total expected intensity an addition spectrum of APP alone and holo-Lf alone spectra was also calculated (blue). **F.** Normalised fluorescence spectra for the addition spectrum (blue) and the spectra of the APP and Lf mixture (red). **G.** The difference in fluorescence intensity between the addition spectrum and the spectra of the mixture of APP and Lf was plotted (Eq 2 in Supp data) as a function of Lf concentration assuming a 2-site model with two different dissociation constants. Data are means ± SEM of 3 experiments performed in triplicate.

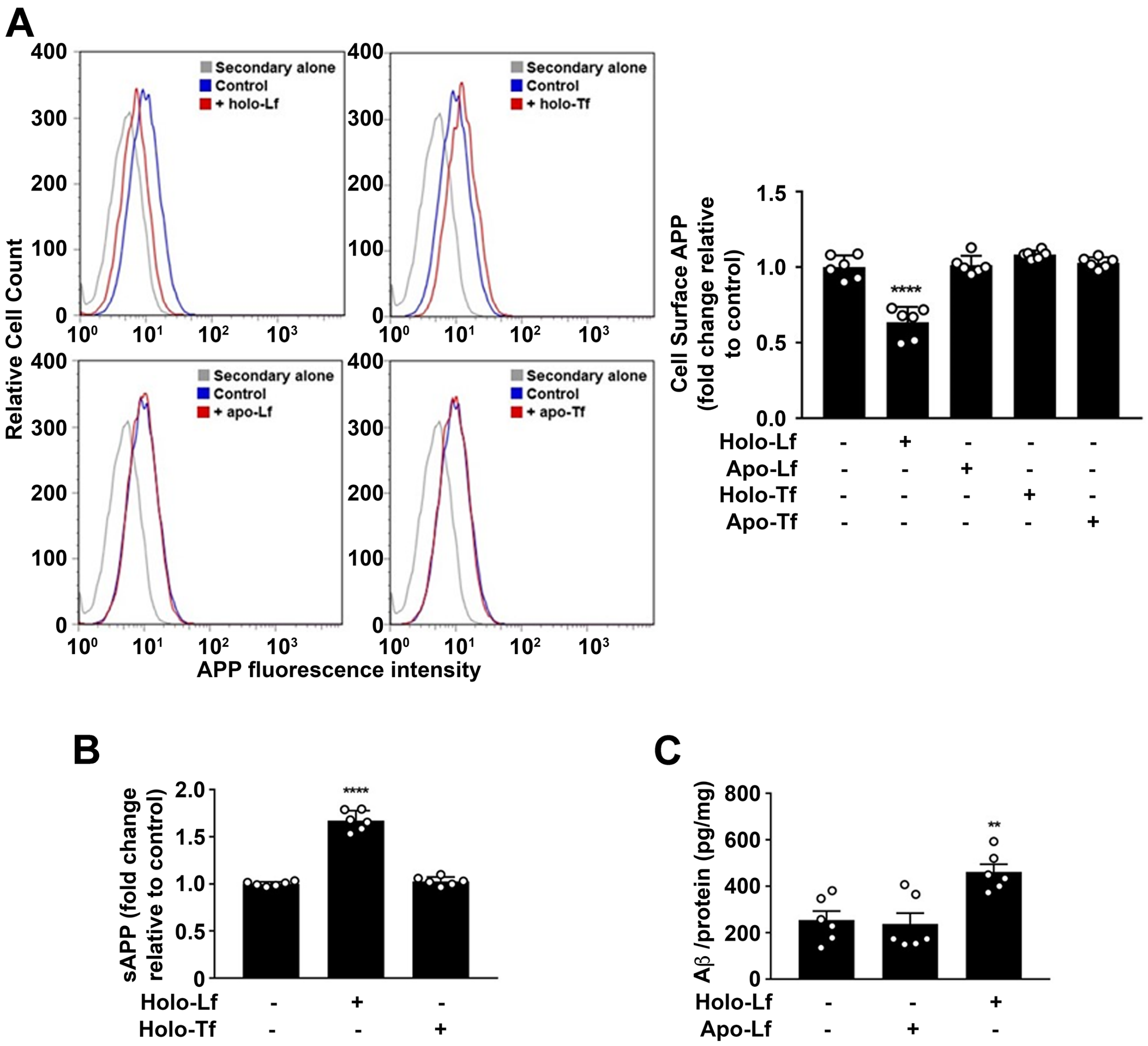

Figure S3. Holo-Lf decreases cell surface APP levels and promotes the amyloidogenic processing of APP. A. Confirmation that only holo-Lf decreases APP levels on the cell surface was performed using FACS on non-permeabilised SH-SY5Y cells to determine cell surface APP (by ab15272) after incubation with holo-Lf, holo-Tf, apo-Lf or apo-Tf (500 nM; 2 h). **B.** Primary murine neurons treated with holo-Lf or holo-Tf (500 nM; 2 h) where evaluated for total sAPP release into the media. **C.** Aβ production was also measured by ELISA on cell lysate after treatment with apo- or holo-Lf (500 nM; 2 h). Data are means ± SEM of 3 experiments performed in duplicate and statistical analysis was by two-tailed t-tests, ** $p < 0.01$ and **** $p < 0.0001$ relative to non-treated controls.

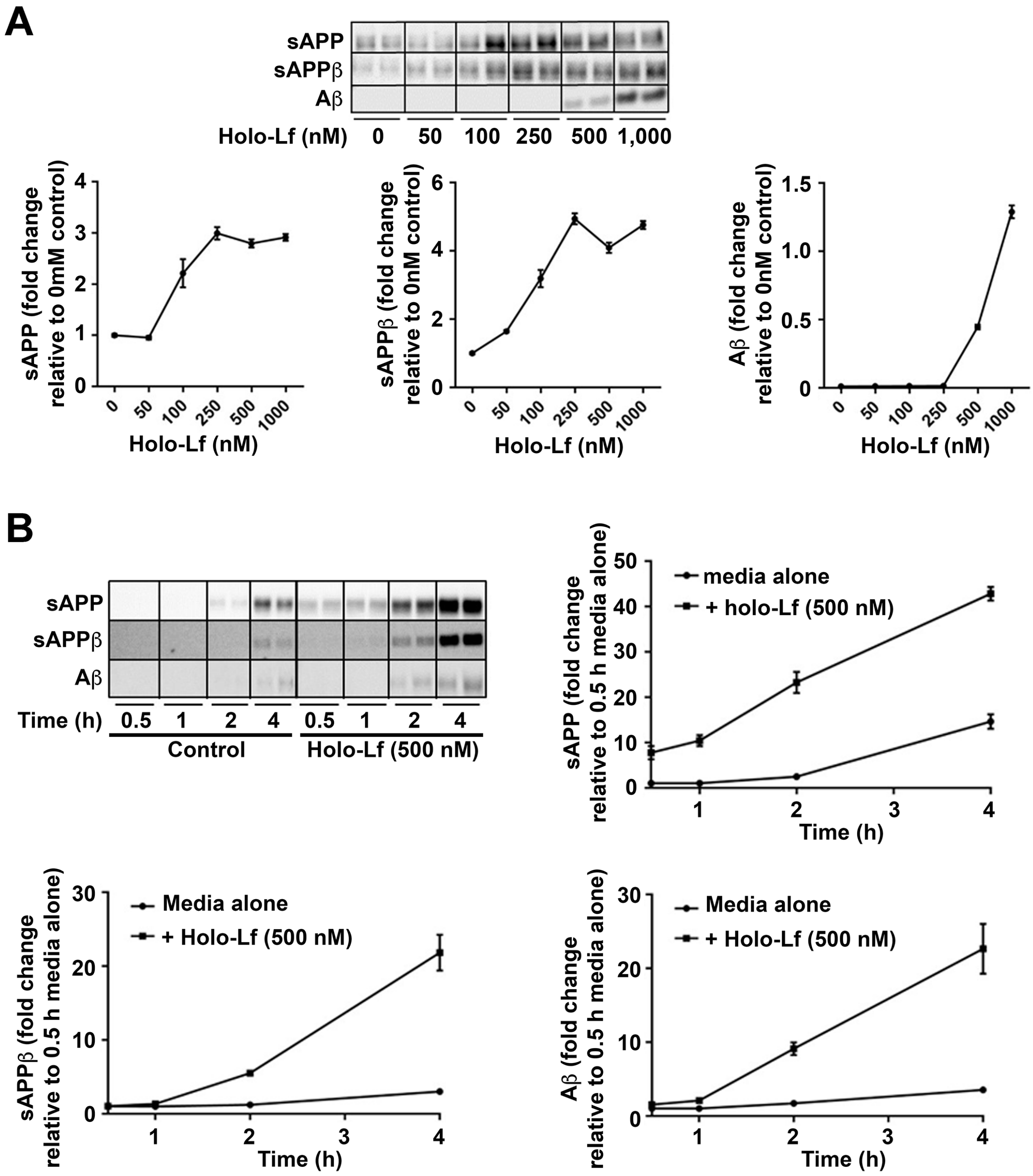

Figure S4. Holo-Lf promotes the amyloidogenic processing of APP. A&B. Comparable changes to total sAPP, sAPPβ and Aβ in media were observed in SH-SY5Y cells exposed to increasing amounts of holo-Lf (0-1000 nM) for 2 h (**A**) or 500 nM holo-Lf at a range of exposure times up to 4 h (**B**). Data are means ± SEM of 3 experiments performed in duplicate.

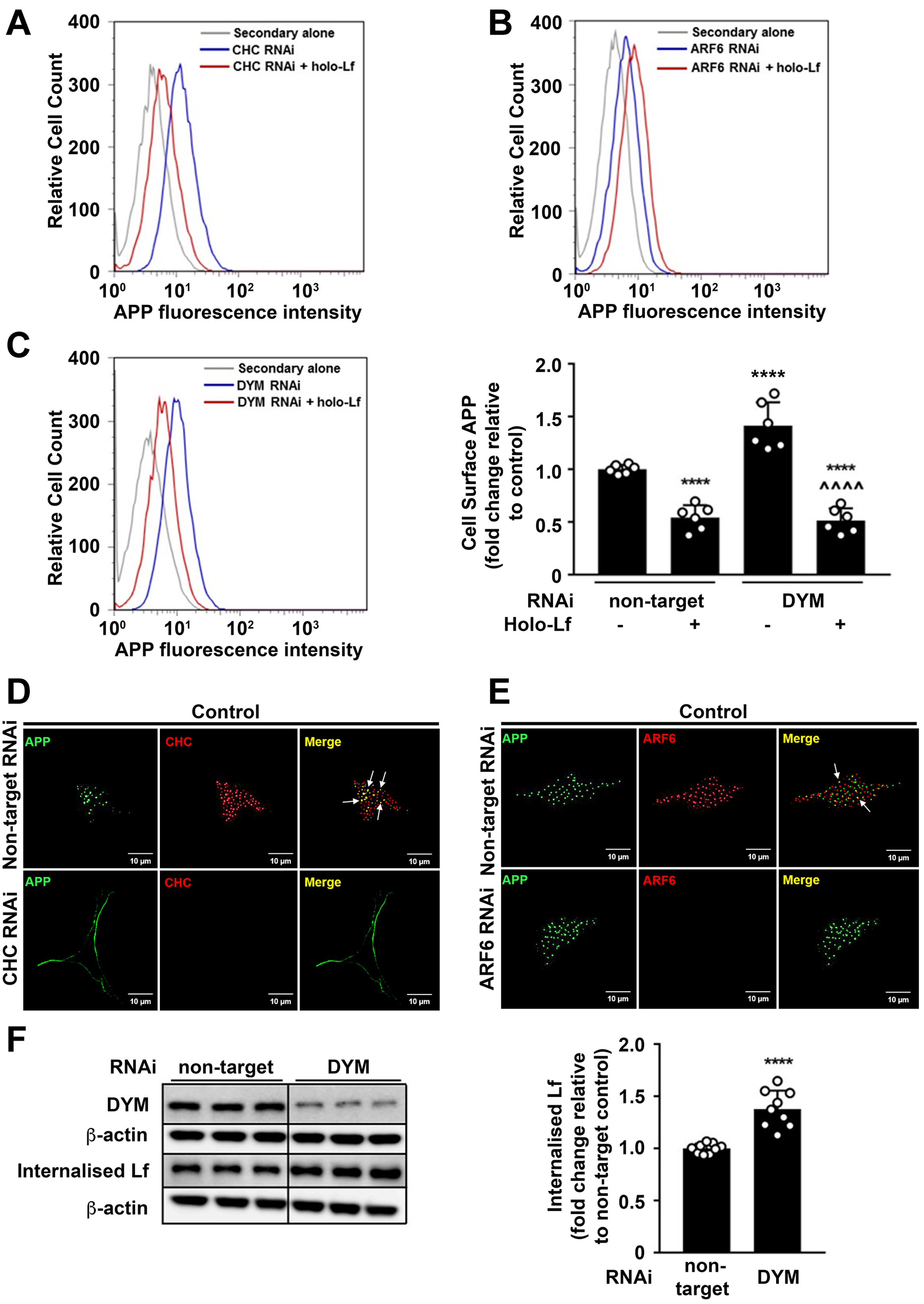

Figure S5. Holo-Lf mediated APP internalisation is clathrin-independent and ARF6-dependent. A-C. FACs histograms for APP levels on the cell surface of non-permeabilised SH-SY5Y cells after being transfected with control non-target and CHC (**A**), ARF6 (**B**) or DYM

(**C**) RNAi (20 nM) for 48 h. Cells were subjected to a 2 h incubation with 500 nM holo-Lf. FACS was performed using ab15272 recognising N-terminal extracellular epitopes of APP. Quantified histogram data of cell surface APP with CHC (**A**), ARF6 (**B**) are shown in Fig. 3, whereas (**C**) shows DYM RNAi depict fold change compared with levels derived from non-targeting control cells. **D&E.** Deconvoluted images of double immunofluorescence confocal microscopy of wt-APP^{695} SH-SY5Ys without holo-Lf treatment. After RNAi depletion of CHC or ARF6 as in **(A&B)**, surface APP was labelled with an APP antibody (22C11) (green) at 4°C before replacing media for 1 h at 37°C. An APP secondary detection antibody was then added with total CHC (ab21679) (red) **(C)** or ARF6 (ab131261) (red) **(D)** co-labelling**.** Co-localisation of APP with CHC **(C)** and ARF6 **(D)** are represented as yellow in the merged image (white arrows). **F.** Effect of DYM RNAi on holo-Lf internalisation in SH-SY5Y cells. SH-SY5Y cells RNAi treated in the same conditions as **(C)** were subjected to the ligand internalisation assay. Total cellular DYM protein expression, and internalised holo-Lf were confirmed by Western blot. **C&F.** Data are means ± SEM of 3 experiments performed at least in duplicate. Statistical analysis was by two-way ANOVA **(C)** or two-tailed t-tests **(D)**, **** $p < 0.0001$ depicts fold change compared to non-targeting control and in **C**, ^^^^ $p < 0.0001$ compares to DYM RNAi without holo-Lf. **D&E.** Images are a representative from multiple cells within experiments carried out in duplicate. Scale bar = 10 µm.

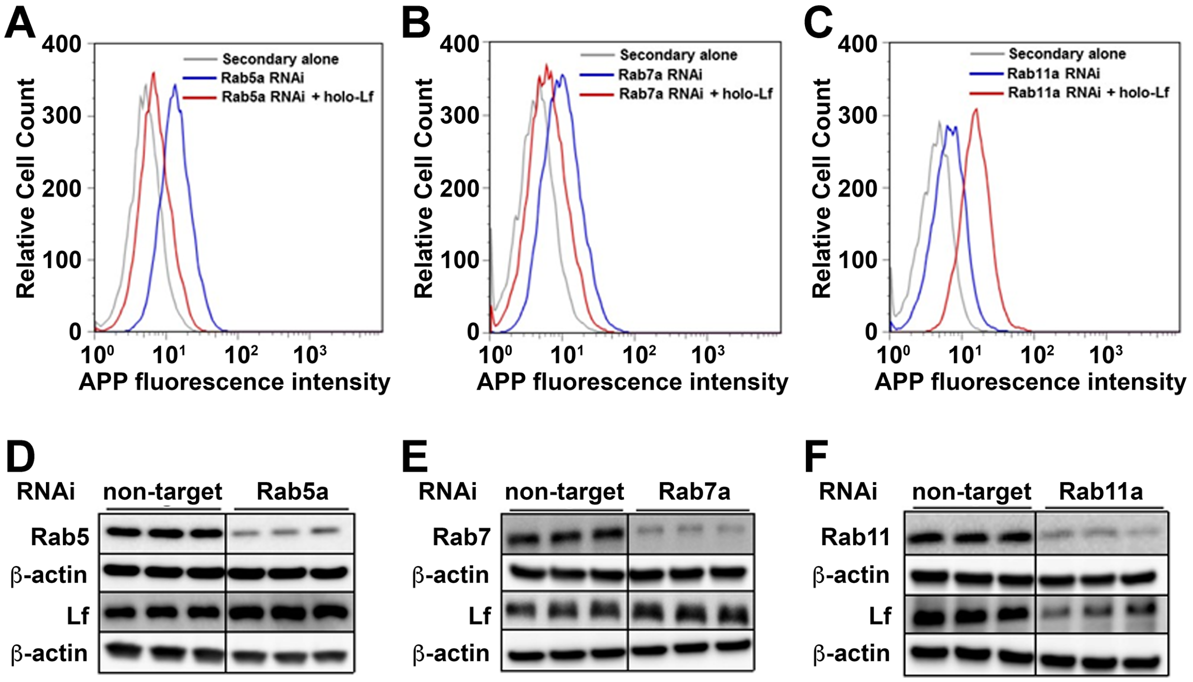

Figure S6. Holo-Lf mediated amyloidogenic processing of APP requires ARF6 but not CHC or DYM. A-C. FACs histogram examples for cell surface APP levels (ab15272) on the cell surface of non-permeabilised SH-SY5Y with and without holo-Lf (500nM; 2 h) after treatment with RNAi (20 nM; 48 h) for Rab5a **(A)**, Rab7a **(B)** Rab11a **(C)** and a non-targeted control. **D-F.** Within the same experimental parameters as **A-C**, the representative Western blots of the effect of Rab5a **(D)**, Rab7a **(E)**, and Rab11a **(F)** knockdown on internalisation of biotinylated holo-Lf (0.5 mg/mL; 1 h at 37°C) as measured by the ligand internalisation assay. Residual surface biotin was stripped with MeSNa so that only internalised biotinylated Lf could be detected in the total cell lysate.

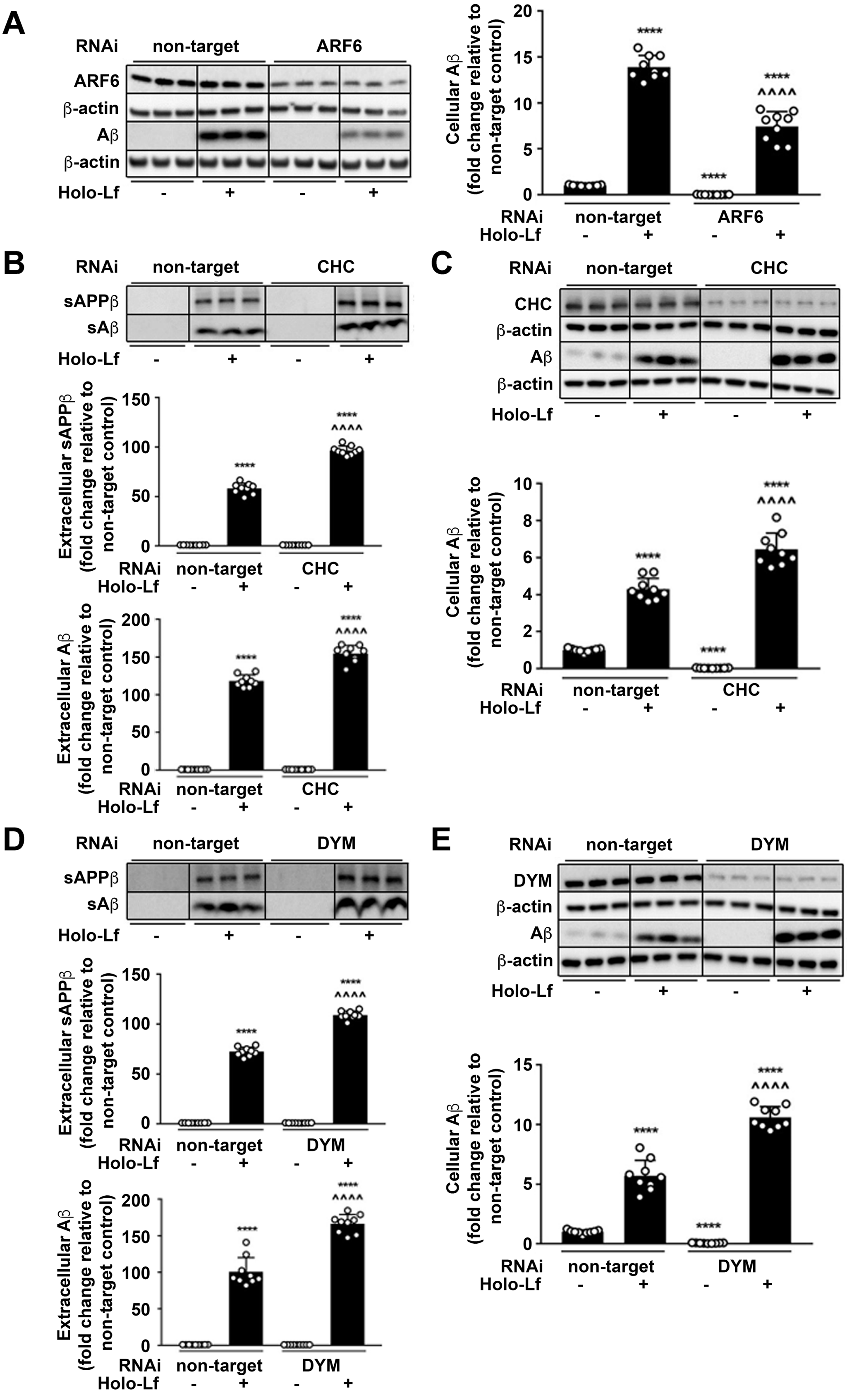

Figure S7. Holo-Lf mediated amyloidogenic processing of APP is clathrin-independent and ARF6-dependent. A. Amyloidogenic processing of APP induced by holo-Lf (500nM; 2 h)

in wtAPP^{695} SH-SY5Y pretreated with control non-target or ARF6 RNAi (20 nM; 48 h) was measured by Aβ production in the cell lysate. **B-E.** Effect of CHC (40 nM; 72 h) **(B & C)** and DYM (20 nM; 48 h) **(D & E)** RNAi on the amyloidogenic processing of APP in media (**B & D**) and cell lysate (**C & E**) in SH-SY5Y-APP695 cells exposed to holo-Lf (500nM; 2 h). Total cellular CHC **(C)** and DYM **(E)** protein expression was confirmed. Protein levels quantified by densitometry and normalised against β-actin content are means ± SEM of 3 experiments performed in triplicate. Statistical analysis by two-way ANOVA depicts fold change compared with control non-target cells, **** $p < 0.0001$ or holo-Lf treated non-targeting cells, ^^^^ $p < 0.0001$.

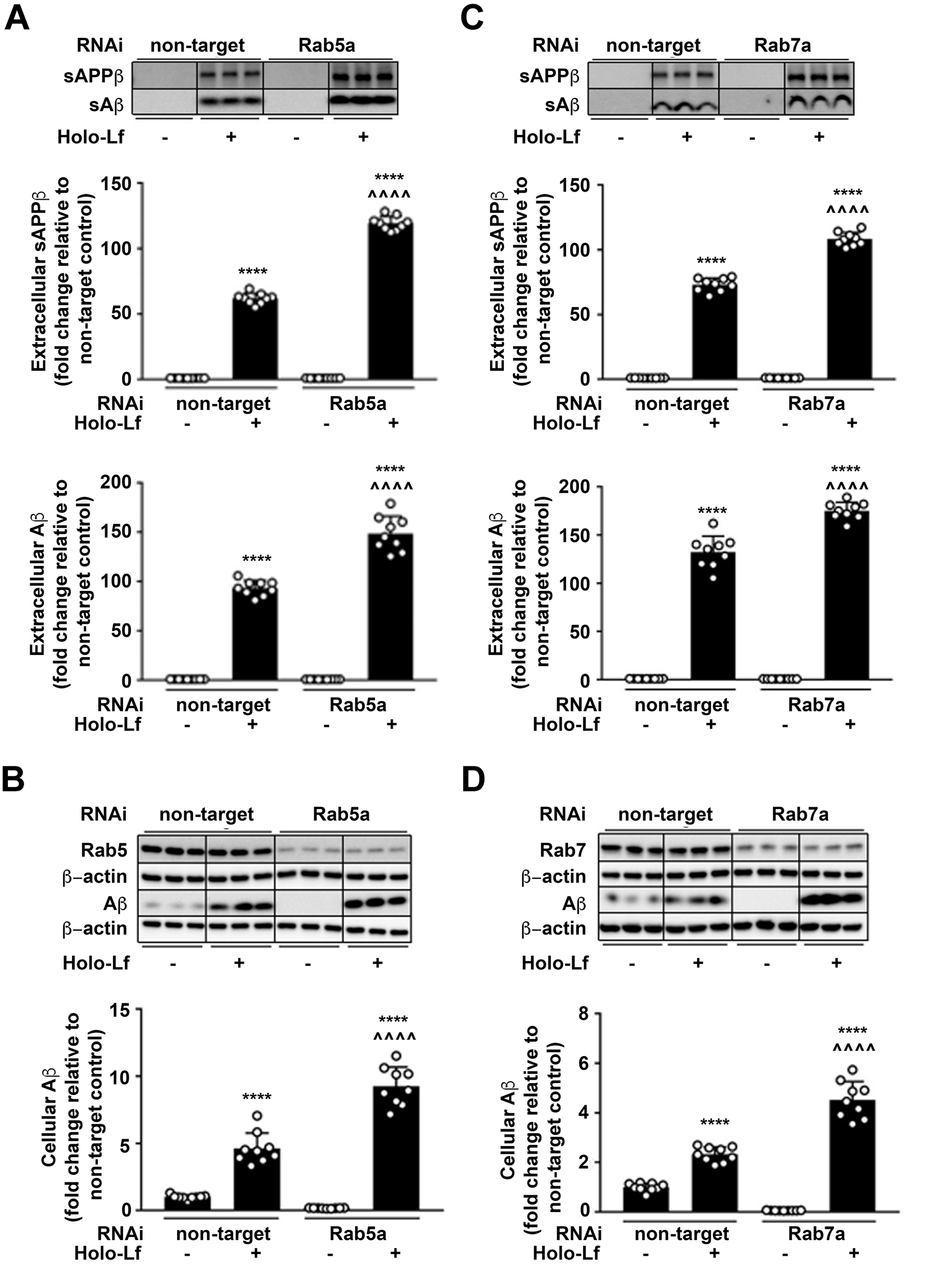

Figure S8. Holo-Lf mediated amyloidogenic processing of APP requires Rab11a but not Rab5a or Rab7a. A-D. Effect of Rab5a (20 nM; 48 h) **(A & B)** and DYM (20 nM; 48 h) **(C & D)** RNAi on the amyloidogenic processing of APP in media (**A & C**) and cell lysate (**B & D**) in SH-SY5Y-APP695 cells exposed to holo-Lf (500nM; 2 h). Total cellular Rab5a **(B)** and Rab7a

(D) protein expression was confirmed. Data are means ± SEM of 3 experiments performed in triplicate and normalised to total protein **(A & C)** or β-actin **(B & D)**. Statistical analysis was by two-way ANOVA, **** $p < 0.0001$ depicts fold change compared to non-targeting control and ^^^^ $p < 0.0001$ to holo-Lf treated non-targeting cells.

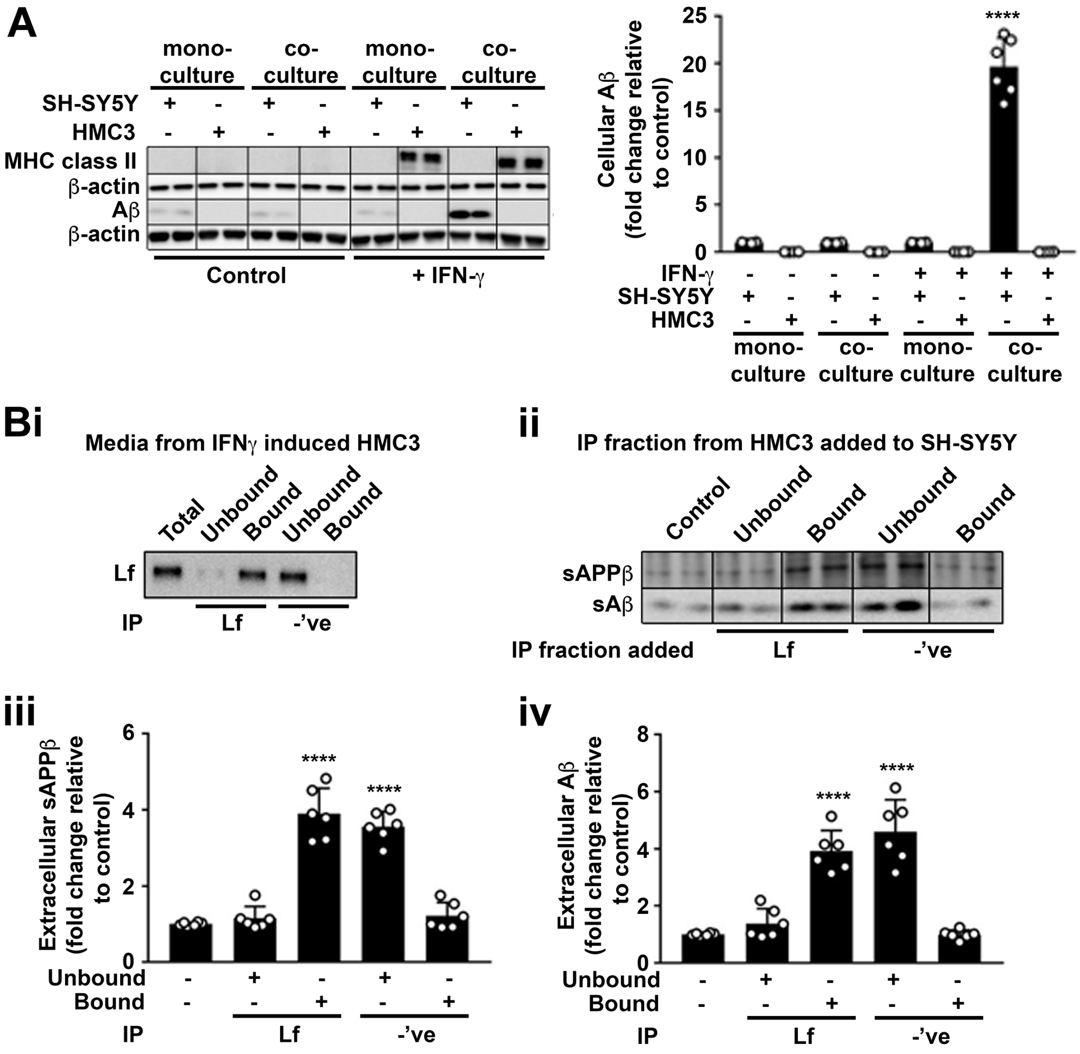

Figure S9. Secreted holo-Lf from activated microglia reduces neuronal surface presented APP and increases APP amyloidogenic processing. A. In monocultures and a transwell co-culture with HMC3 microglia cultured in the upper inserts and wtAPP^{695} SH-SY5Y in the lower wells, human recombinant IFN-γ (10 ng/ml; 24 h) was used to activate microglia, as confirmed by the MHC class II marker. IFN-γ induced changes in cellular Aβ was quantified. **B.** Immunoprecipitation was used to remove Lf from the media of HMC3 cells activated with IFN-γ (10 ng/ml; 24 h) with an antibody against β-actin as a negative control (**i**). The capture antibody confirmed specificity of interaction. Eluted fractions (bound) as well as the media flow through (unbound) of both the Lf and negative control immunoprecipitates (**ii**) were then applied to wtAPP^{695} SH-SY5Y monocultures and incubated for 2 h to determine sAPPβ (**iii**) and Aβ (**iv**) secretion into the media. Data are mean ± SEM of 3 experiments performed in duplicate as a minimum and normalised against a control protein. Statistical analysis was by two-tailed t-tests, **** $p < 0.0001$ depicts a fold change compared to control conditions (without IFN-γ) in the same cell model (**A**) or no treatment (**B**).

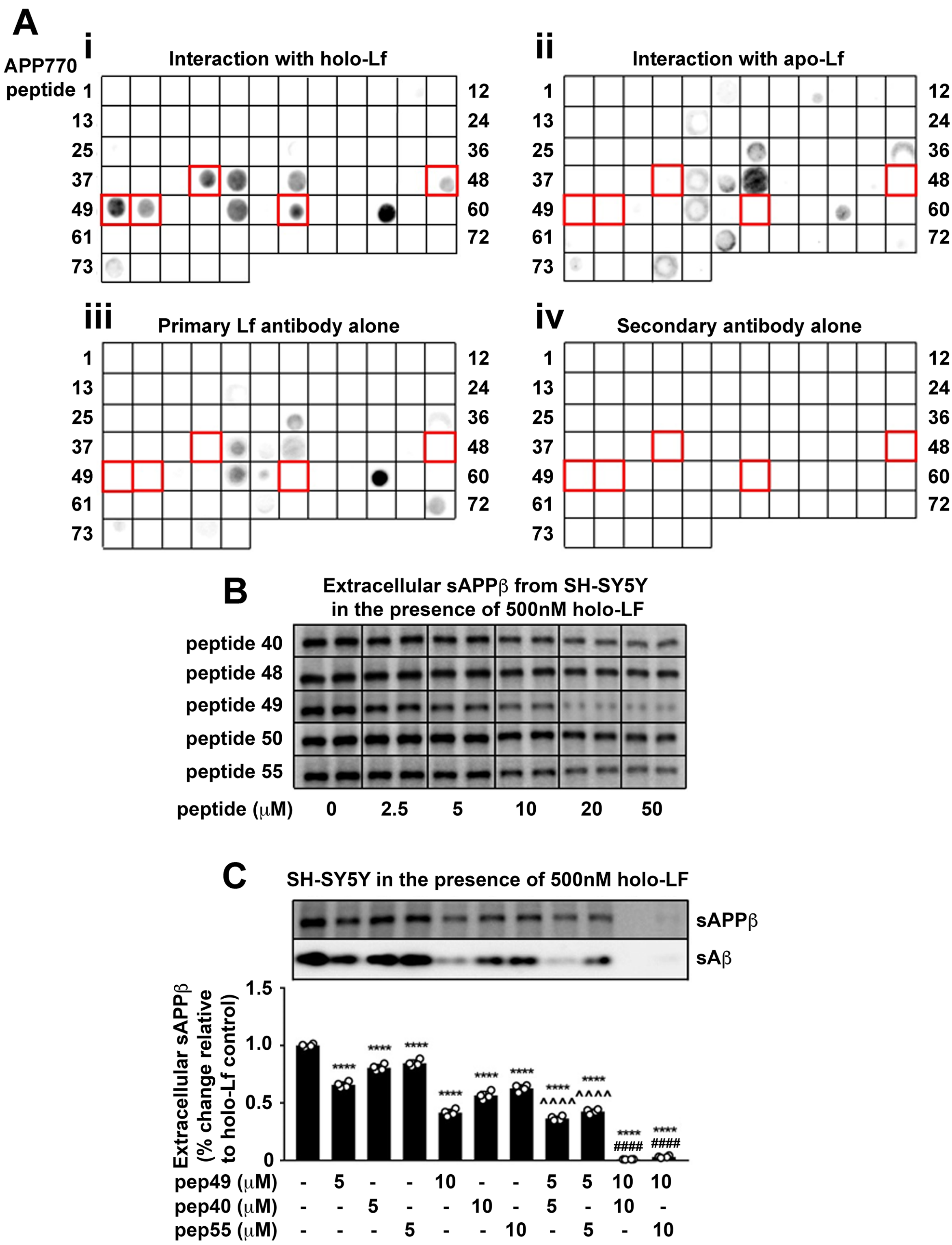

Figure S10. Identification of the holo-Lf binding sites on APP required to holo-Lf induced amyloidogenic processing of APP. A. A peptide array consisting of 15-mer peptides with a 5 amino acid overlap at each end for full length human APP^{770} protein was probed with holo-Lf (**i**) as well as the control apo-Lf (**ii**), anti-Lf primary antibody only (**iii**) and anti-rabbit secondary antibody only (**iv**) to determine holo-Lf specificity (highlighted as red

squares). **B.** Western blot analysis on sAPPβ formation corresponding to Fig. 7C. **C.** Western blot analysis on sAβ (quantified in Fig. 7D) and sAPPβ after APP peptide 40, 49 and 55 were used for combinatory inhibition of holo-Lf induced amyloidogenic processing of APP from wtAPP^{695} SH-SY5Y. Data is mean ± SEM of 2 experiments performed in duplicate with statistical analysis by two-way ANOVA comparing holo-Lf treated control, **** $p < 0.0001$, 5 μM peptide 49 alone, ^^^^ $p < 0.0001$, or 10μM peptide 49 alone, $^{\#\#\#\#}$ $p < 0.0001$.

SUPPLEMENTAL TABLES

Tables S1. Full Boruta analysis of ROSMAP dataset

A. Full gene list of Boruta analysis for disease classification with DEGs highlighted.
B. Full gene list of Boruta analysis for amyloid prediction in 7 cortical regions and the hippocampus
C. Full gene list of Boruta analysis for amyloid prediction in superior frontal gyrus
D. Full gene list of Boruta analysis for phosphorylated tau prediction in 7 cortical regions and the hippocampus
E. Full gene list of Boruta analysis for phosphorylated tau prediction in superior frontal gyrus

Attached Excel spreadsheet

Tables S2. Top 20 ranked genes for disease classification

A. Top 20 ranked genes for disease classification by 'xgbtree' modeling.
B. Top 20 ranked genes for disease classification by 'RandomForest' modeling.
C. Top 20 ranked genes for disease classification by 'Glmnet' modeling.
D. Top 20 ranked genes for disease classification by 'Rpart' modeling.

Attached Excel spreadsheet

Table S3. Differentially expressed genes in AD cases relative to controls ranked by false discovery rate (FDR).

Attached Excel spreadsheet

Table S4. Significantly enriched Gene Ontology biological process categories in the differentially expressed gene list

Attached Excel spreadsheet

Table S5. KEGG pathway analysis of the differentially expressed gene list

Attached Excel spreadsheet

Table S6. Open target analysis of the differentially expressed gene list

Attached Excel spreadsheet

Table S7. Dissociation constants determined from Fluorescence and sedimentation velocity analysis.

	Sedimentation velocity analysis	Fluorescence analysis
$Kd_{1:1}$ (μM)	0.62	0.69
$Kd_{1:2}$ (μM)	8.2	9.4

Table S8. Peptide array consisting of 15-mer peptides with a 5 αα overlap covering the full length APP770 protein sequence indicating 15-mer peptides with a 5 αα overlap that were used to identify interacting sites for holo-Lf in Fig. 8A.

peptide_21	AEEDDSDVWWGGADT	peptide_41	KHRERMSQVMREWEE	peptide_61	KTTVELLPVNGEFSL
peptide_22	GGADTDYADGSEDKV	peptide_42	REWEEAERQAKNLPK	peptide_62	GEFSLDDLQPWHSFG
peptide_23	SEDKVVEVAEEEEVA	peptide_43	KNLPKADKKAVIQHF	peptide_63	WHSFGADSVPANTEN
peptide_24	EEEVAEVEEEEADDD	peptide_44	VIQHFQEKVESLEQE	peptide_64	ANTENEVEPVDARPA
peptide_25	EADDDEDDEDGDEVE	peptide_45	SLEQEAANERQQLVE	peptide_65	DARPAADRGLTTRPG
peptide_26	GDEVEEEAEEPYEEA	peptide_46	QQLVETHMARVEAML	peptide_66	TTRPGSGLTNIKTEE
peptide_27	PYEEATERTTSIATT	peptide_47	VEAMLNDRRRLALEN	peptide_67	IKTEEISEVKMDAEF
peptide_28	SIATTTTTTTESVEE	peptide_48	LALENYITALQAVPP	peptide_68	MDAEFRHDSGYEVHH
peptide_29	ESVEEVVREVCSEQA	peptide_49	QAVPPRPRHVFNMLK	peptide_69	YEVHHQKLVFFAEDV
peptide_30	CSEQAETGPCRAMIS	peptide_50	FNMLKKYVRAEQKDR	peptide_70	FAEDVGSNKGAIIGL
peptide_31	RAMISRWYFDVTEGK	peptide_51	EQKDRQHTLKHFEHV	peptide_71	AIIGLMVGGVVIATV
peptide_32	VTEGKCAPFFYGGCG	peptide_52	HFEHVRMVDPKKAAQ	peptide_72	VIATVIVITLVMLKK
peptide_33	YGGCGGNRNNFDTEE	peptide_53	KKAAQIRSQVMTHLR	peptide_73	VMLKKKQYTSIHHGV
peptide_34	FDTEEYCMAVCGSAM	peptide_54	MTHLRVIYERMNQSL	peptide_74	IHHGVVEVDAAVTPE
peptide_35	CGSAMSQSLLKTTQE	peptide_55	MNQSLSLLYNVPAVA	peptide_75	AVTPEERHLSKMQQN
peptide_36	KTTQEPLARDPVKLP	peptide_56	VPAVAEEIQDEVDEL	peptide_76	KMQQNGYENPTYKFF
peptide_37	PVKLPTTAASTPDAV	peptide_57	EVDELLQKEQNYSDD	peptide_77	TYKFFEQMQN
peptide_38	TPDAVDKYLETPGDE	peptide_58	NYSDDVLANMISEPR		
peptide_39	TPGDENEHAHFQKAK	peptide_59	ISEPRISYGNDALMP		
peptide_40	FQKAKERLEAKHRER	peptide_60	DALMPSLTETKTTVE		

REFERENCES

1. Cappai R, Mok SS, Galatis D, Tucker DF, Henry A, Beyreuther K *et al.* Recombinant human amyloid precursor-like protein 2 (APLP2) expressed in the yeast Pichia pastoris can stimulate neurite outgrowth. *FEBS letters* 1999; **442**(1)**:** 95-98.

2. Henry A, Masters CL, Beyreuther K, Cappai R. Expression of Human Amyloid Precursor Protein Ectodomains inPichia pastoris:Analysis of Culture Conditions, Purification, and Characterization. *Protein Expression and Purification* 1997; **10**(2)**:** 283-291.

3. Blackberg L, Hernell O. Isolation of lactoferrin from human whey by a single chromatographic step. *FEBS letters* 1980; **109**(2)**:** 180-183.

4. Dam J, Velikovsky CA, Mariuzza RA, Urbanke C, Schuck P. Sedimentation Velocity Analysis of Heterogeneous Protein-Protein Interactions: Lamm Equation Modeling and Sedimentation Coefficient Distributions. *Biophys J* 2005; **89**(1)**:** 619-634.

5. Morabito S, Miyoshi E, Michael N, Swarup V. Integrative genomics approach identifies conserved transcriptomic networks in Alzheimer's disease. *Hum Mol Genet* 2020; **29**(17)**:** 2899-2919.

6. Zhang B, Gaiteri C, Bodea LG, Wang Z, McElwee J, Podtelezhnikov AA *et al.* Integrated systems approach identifies genetic nodes and networks in late-onset Alzheimer's disease. *Cell* 2013; **153**(3)**:** 707-720.

7. Berchtold NC, Coleman PD, Cribbs DH, Rogers J, Gillen DL, Cotman CW. Synaptic genes are extensively downregulated across multiple brain regions in normal human aging and Alzheimer's disease. *Neurobiology of aging* 2013; **34**(6)**:** 1653-1661.
