# Supplementary figures and images for: The acute phase protein lactoferrin is a key feature of Alzheimer’s disease and predictor of Aβ burden through induction of APP amyloidogenic processing
Source: Mol Psychiatry. 2021 Aug 16;26(10):5516–31. doi: 10.1038/s41380-021-01248-1 (PMC8758478; doi:10.1038/s41380-021-01248-1)

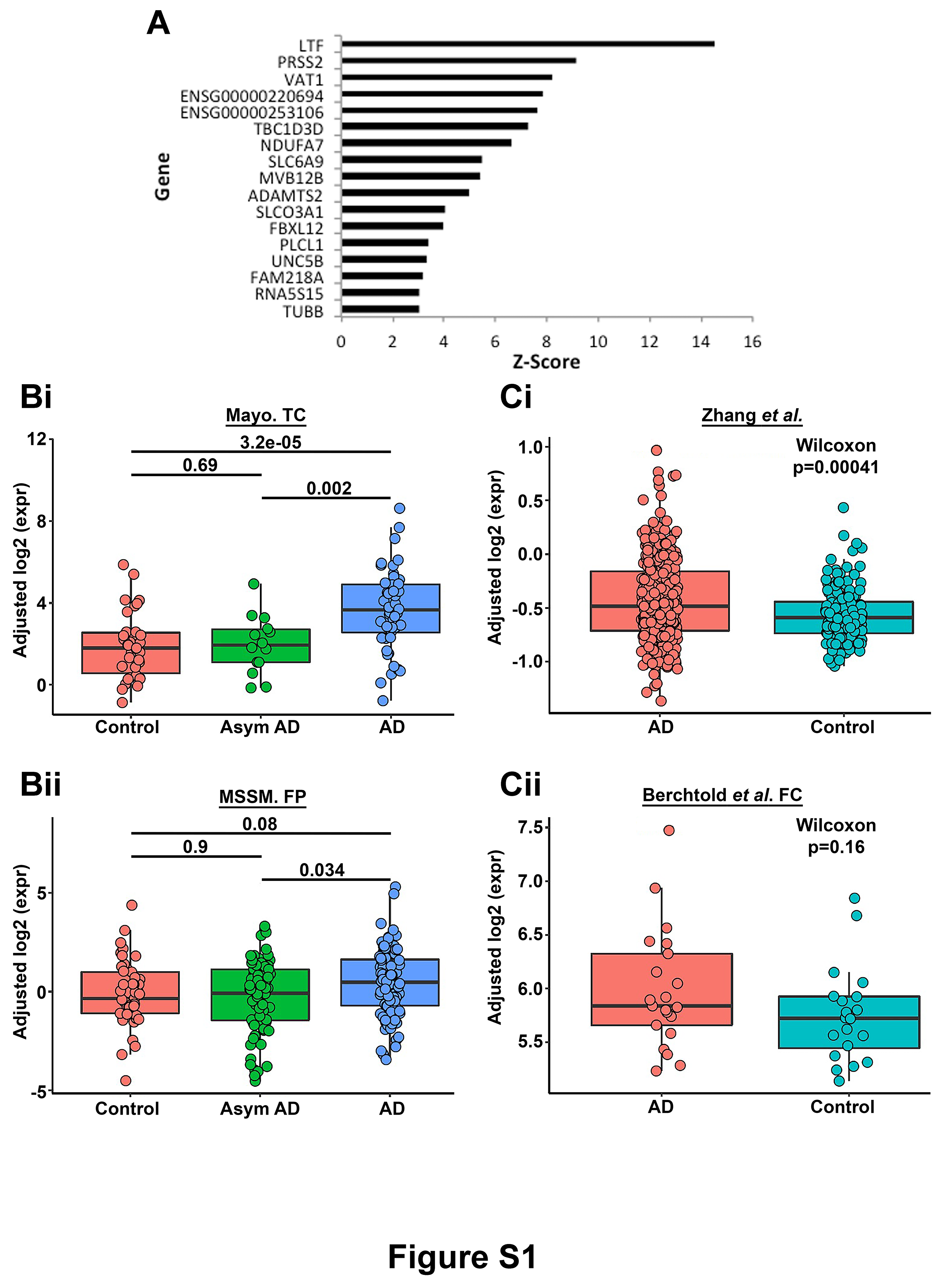

Supplement: Supplementary file 2 — Supplementary Figure 1 [file 41380_2021_1248_MOESM2_ESM.tif]

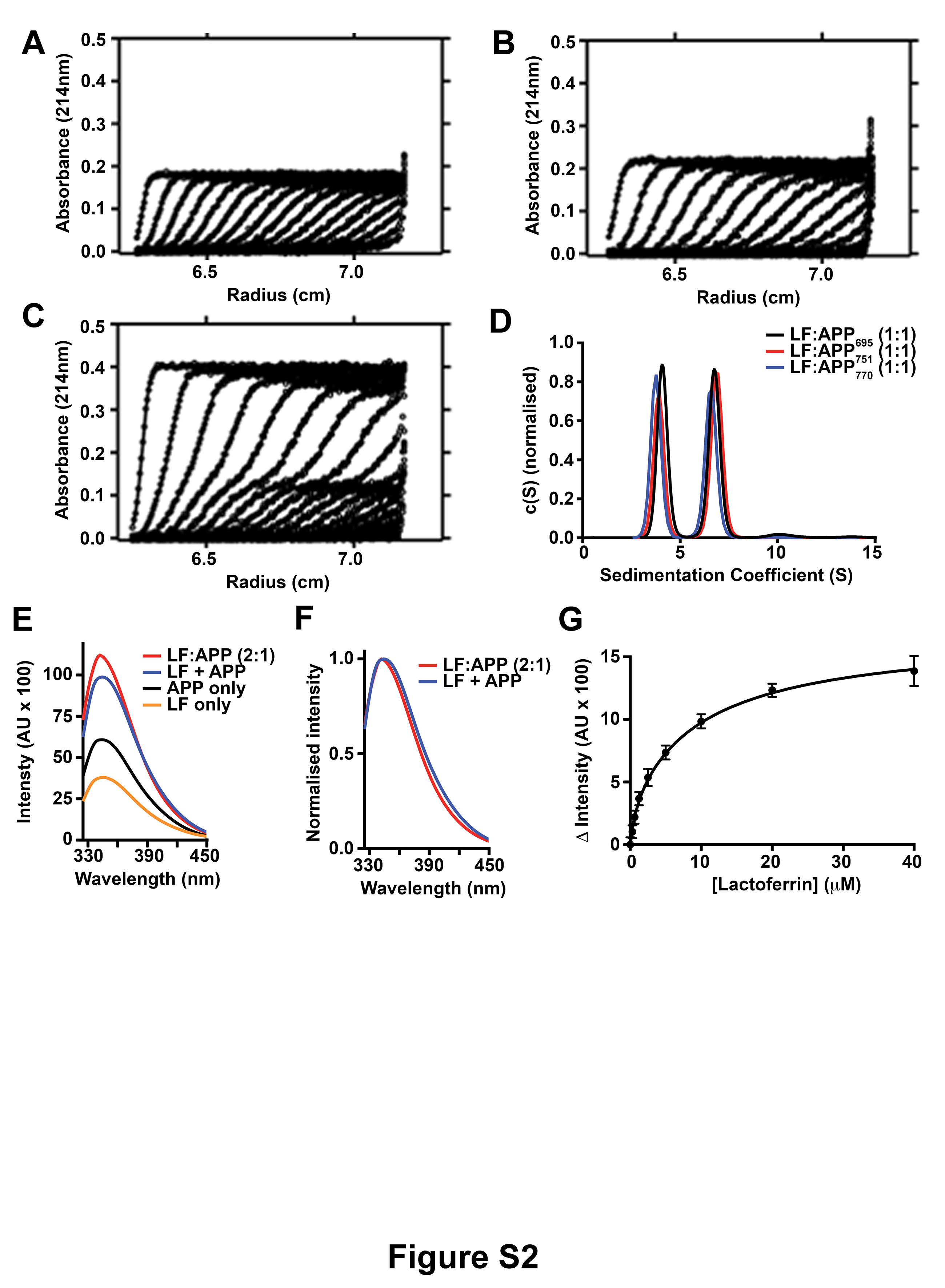

Supplement: Supplementary file 3 — Supplementary Figure 2 [file 41380_2021_1248_MOESM3_ESM.tif]

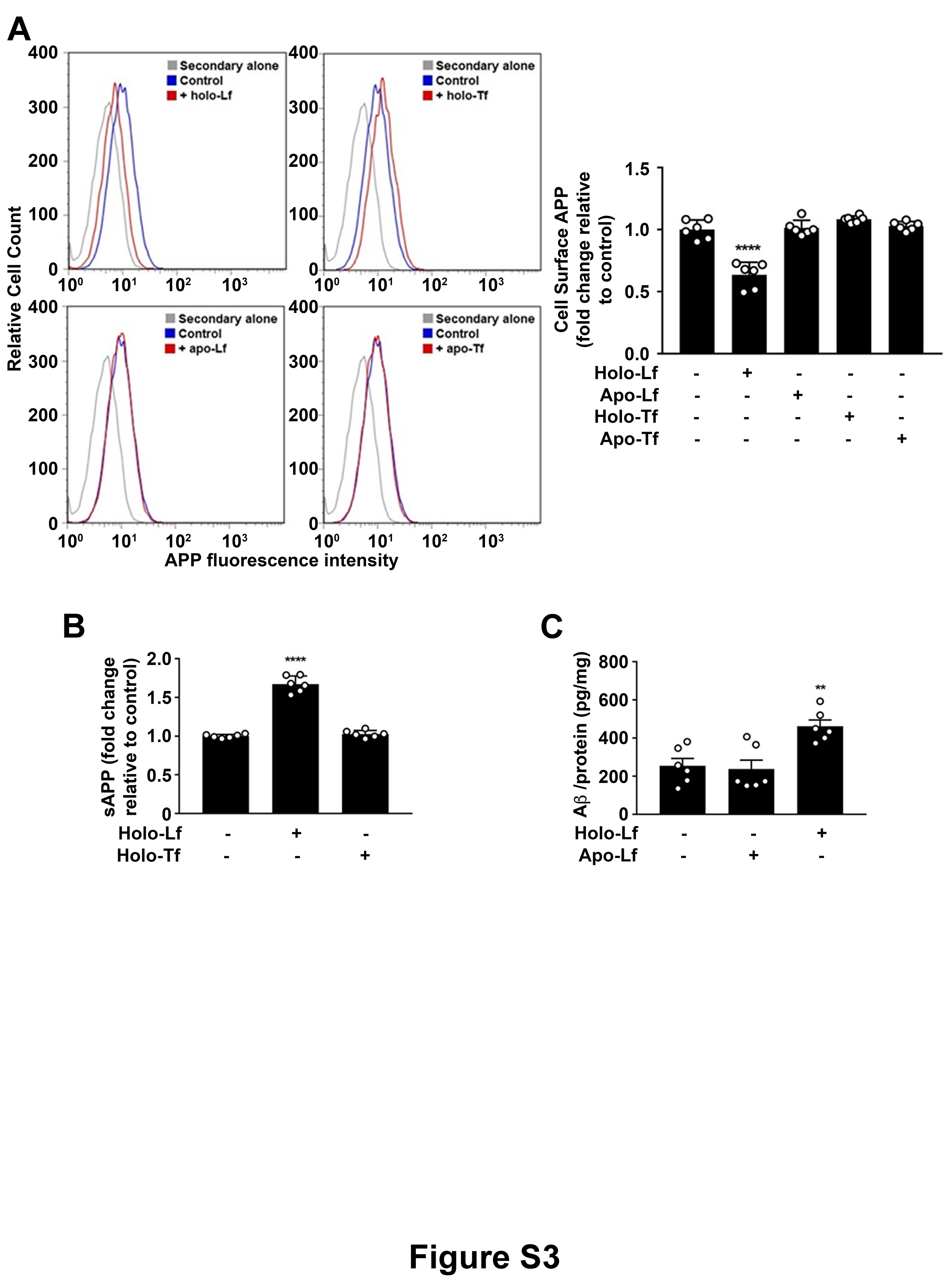

Supplement: Supplementary file 4 — Supplementary Figure 3 [file 41380_2021_1248_MOESM4_ESM.tif]

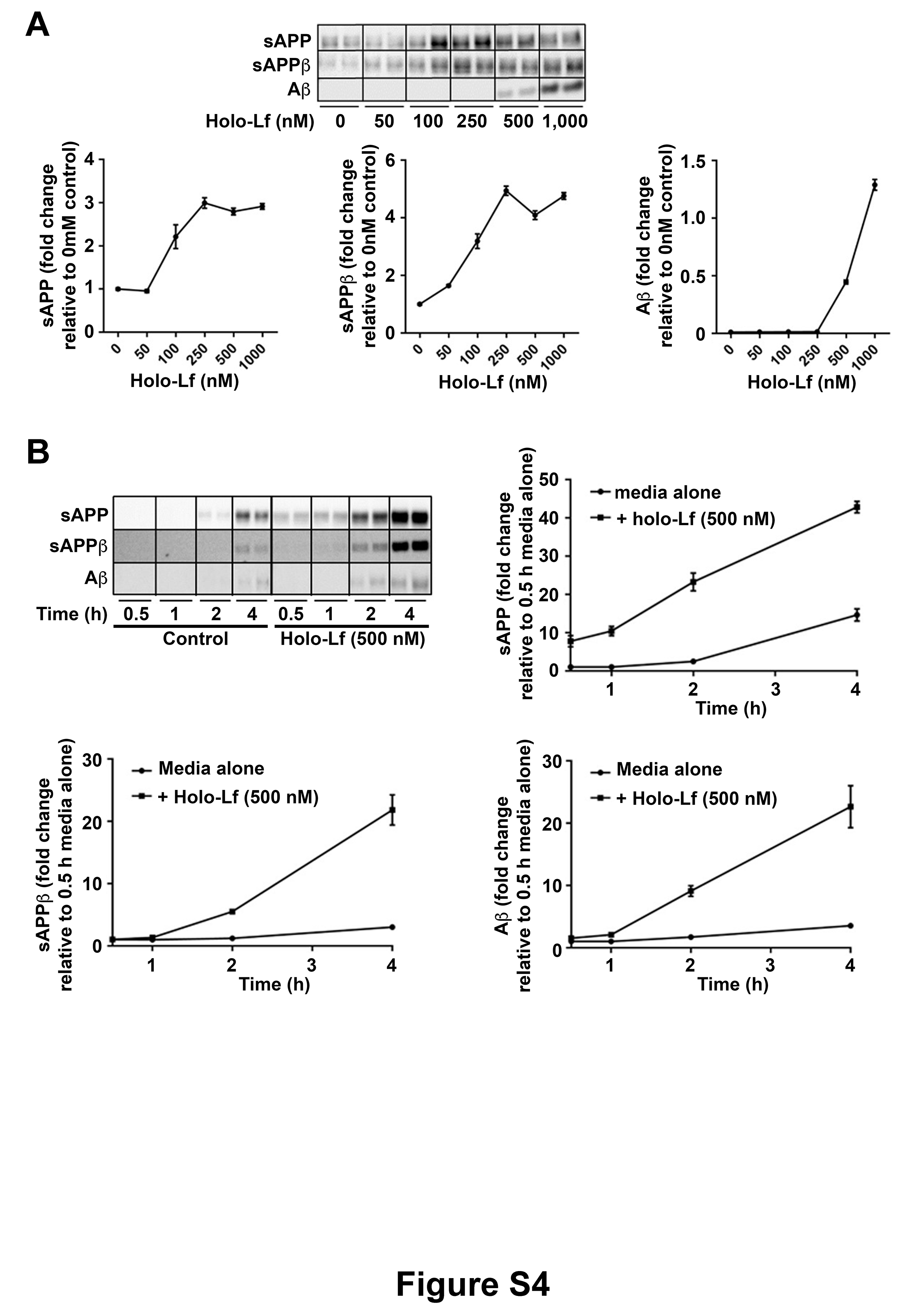

Supplement: Supplementary file 5 — Supplementary Figure 4 [file 41380_2021_1248_MOESM5_ESM.tif]

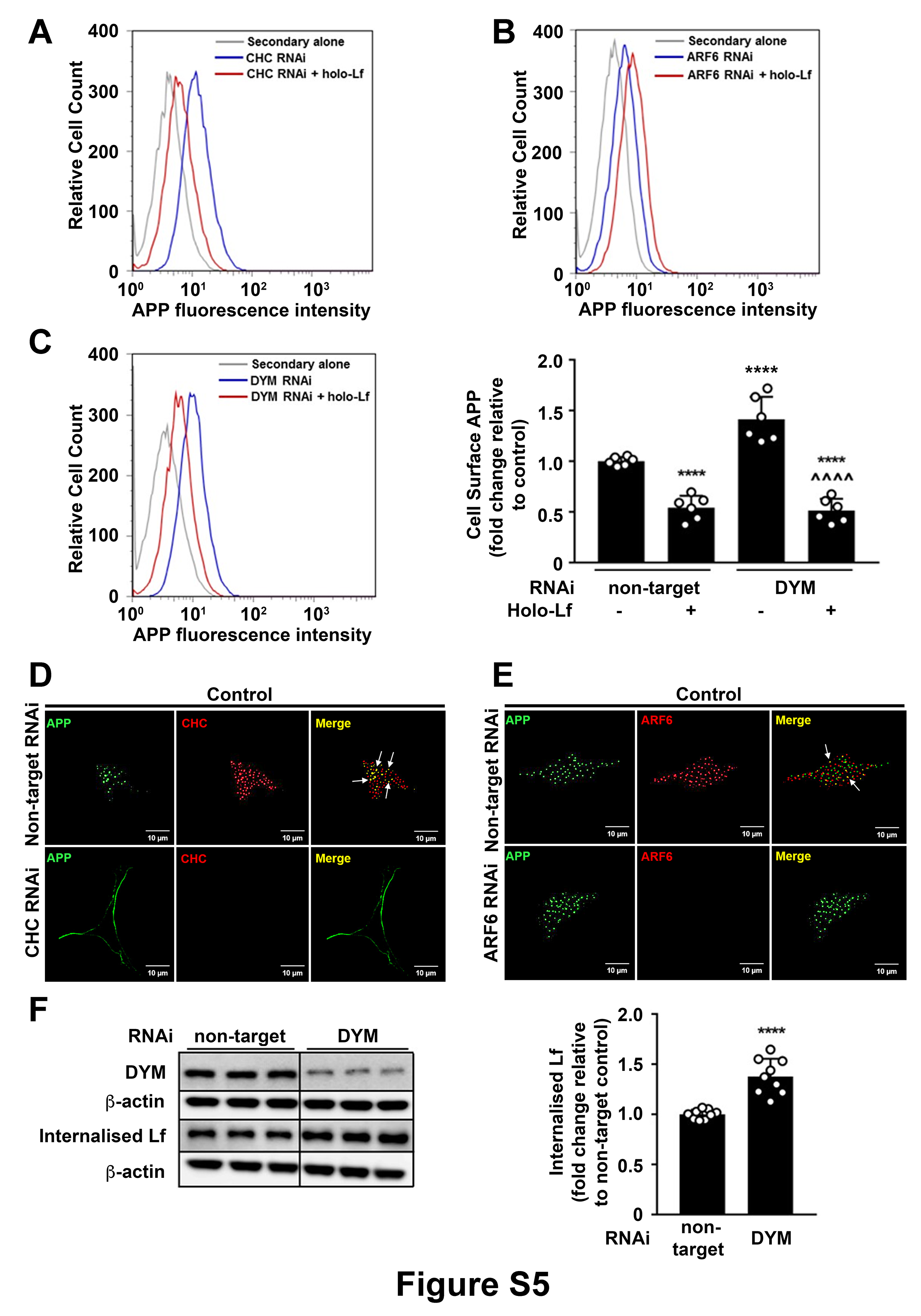

Supplement: Supplementary file 6 — Supplementary Figure 5 [file 41380_2021_1248_MOESM6_ESM.tif]

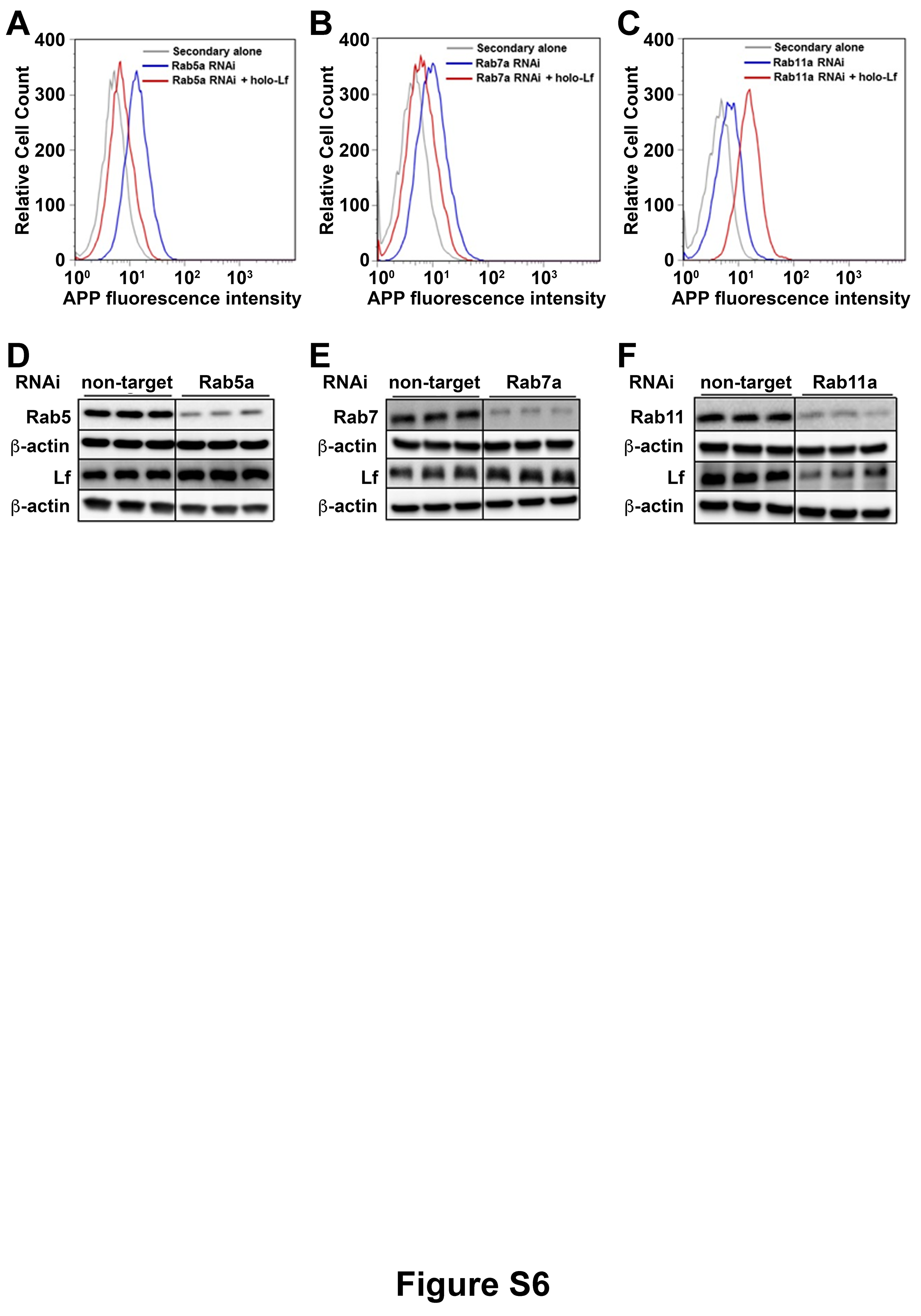

Supplement: Supplementary file 7 — Supplementary Figure 6 [file 41380_2021_1248_MOESM7_ESM.tif]

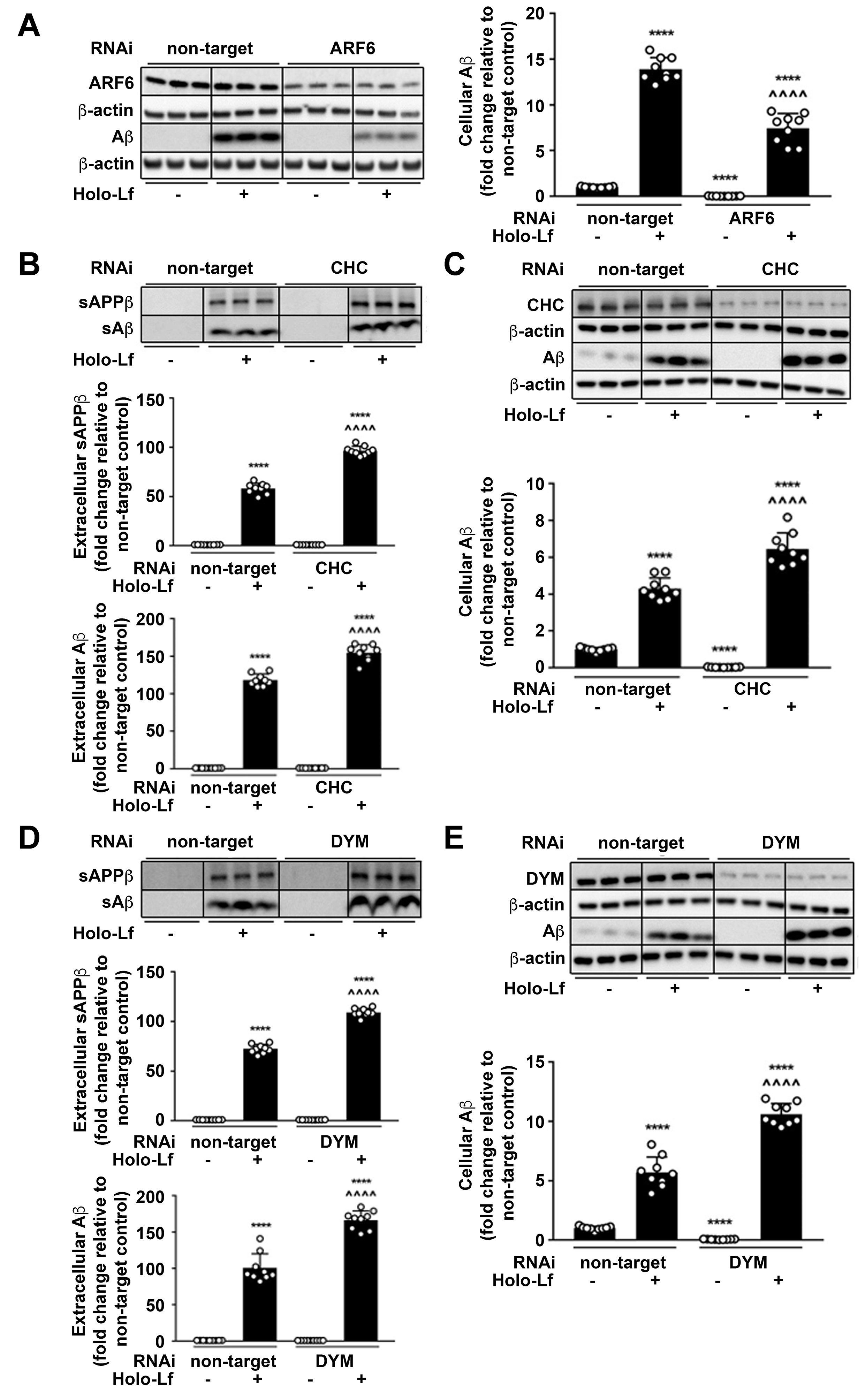

Supplement: Supplementary file 8 — Supplementary Figure 7 [file 41380_2021_1248_MOESM8_ESM.tif]

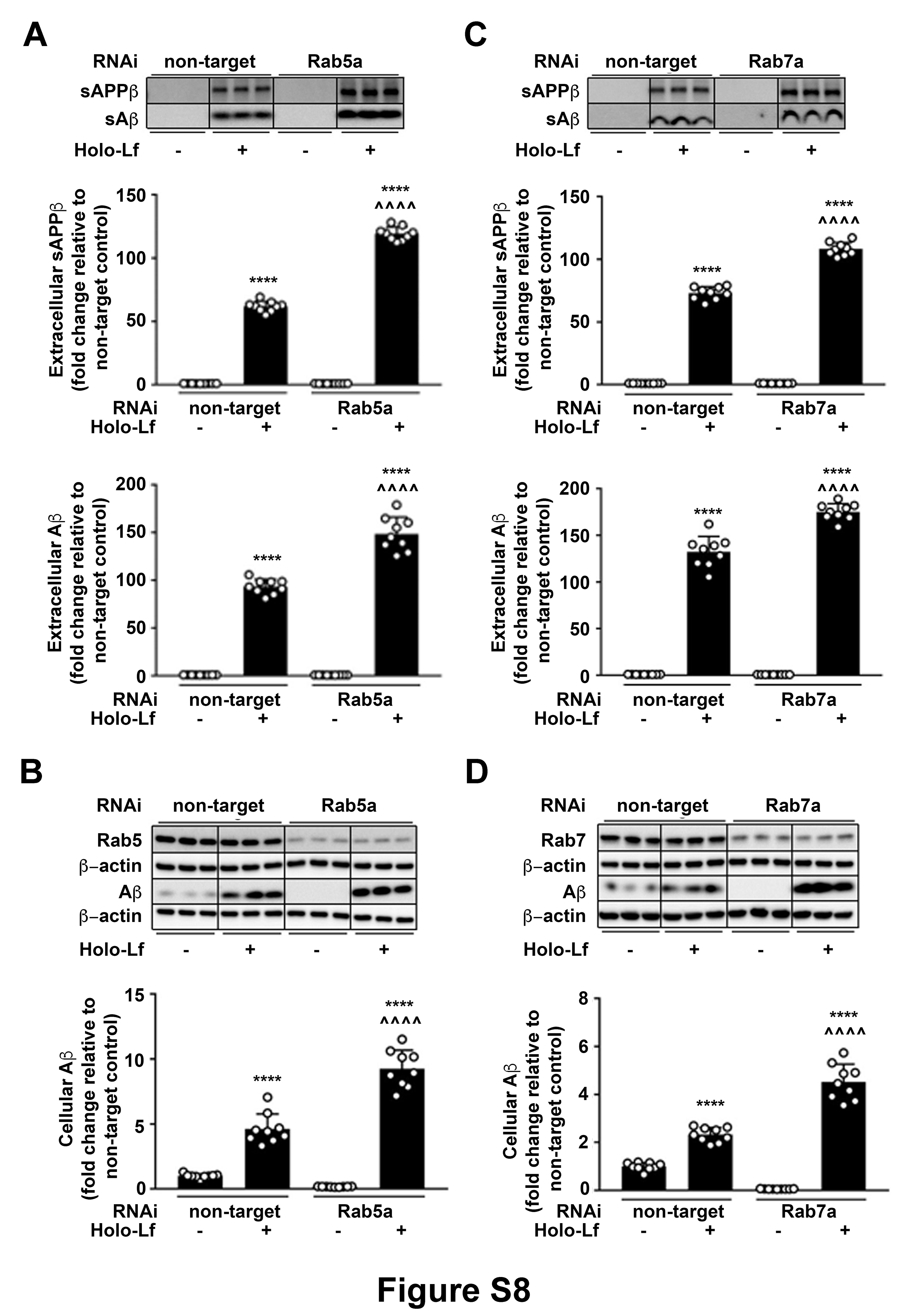

Supplement: Supplementary file 9 — Supplementary Figure 8 [file 41380_2021_1248_MOESM9_ESM.tif]

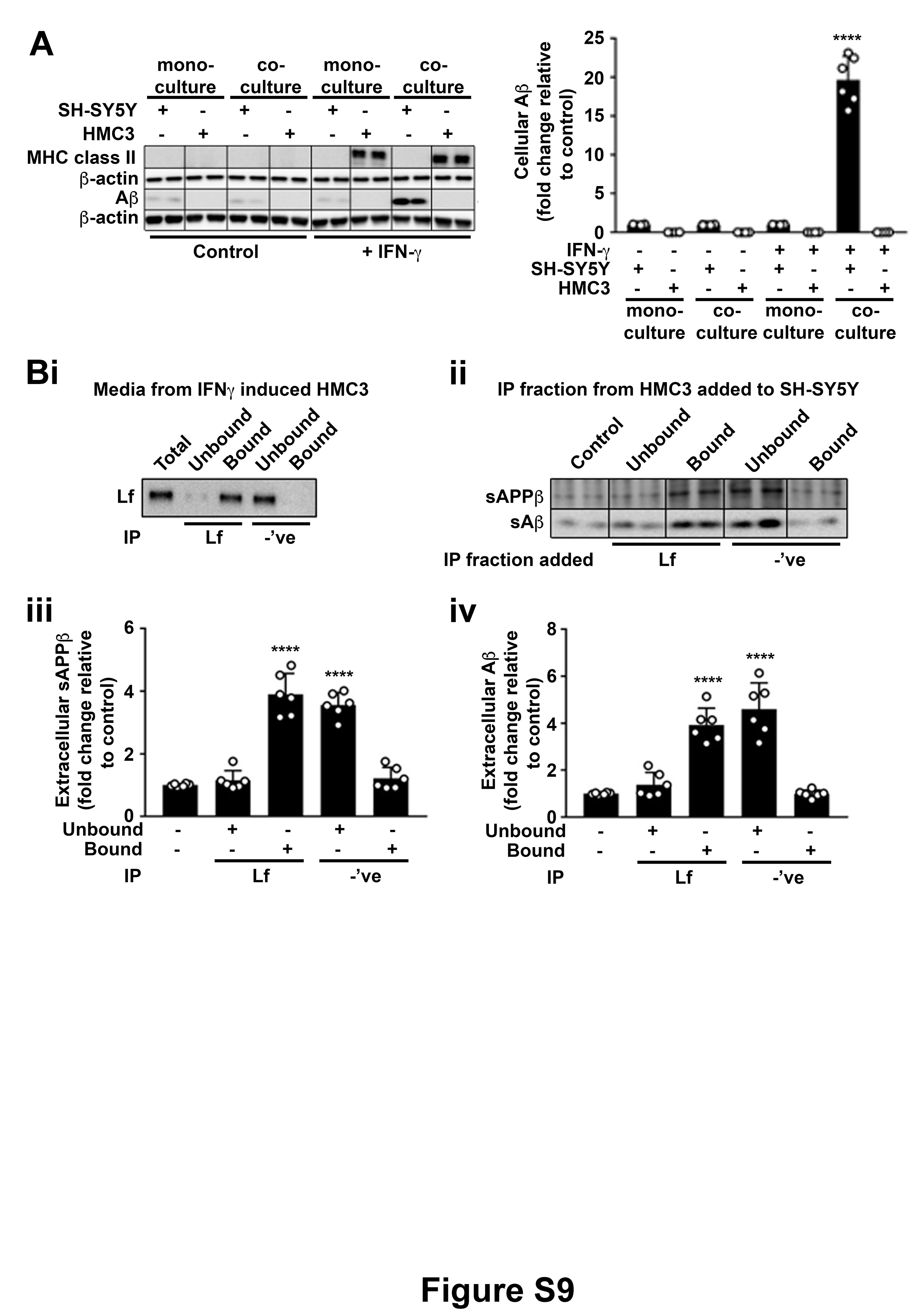

Supplement: Supplementary file 10 — Supplementary Figure 9 [file 41380_2021_1248_MOESM10_ESM.tif]

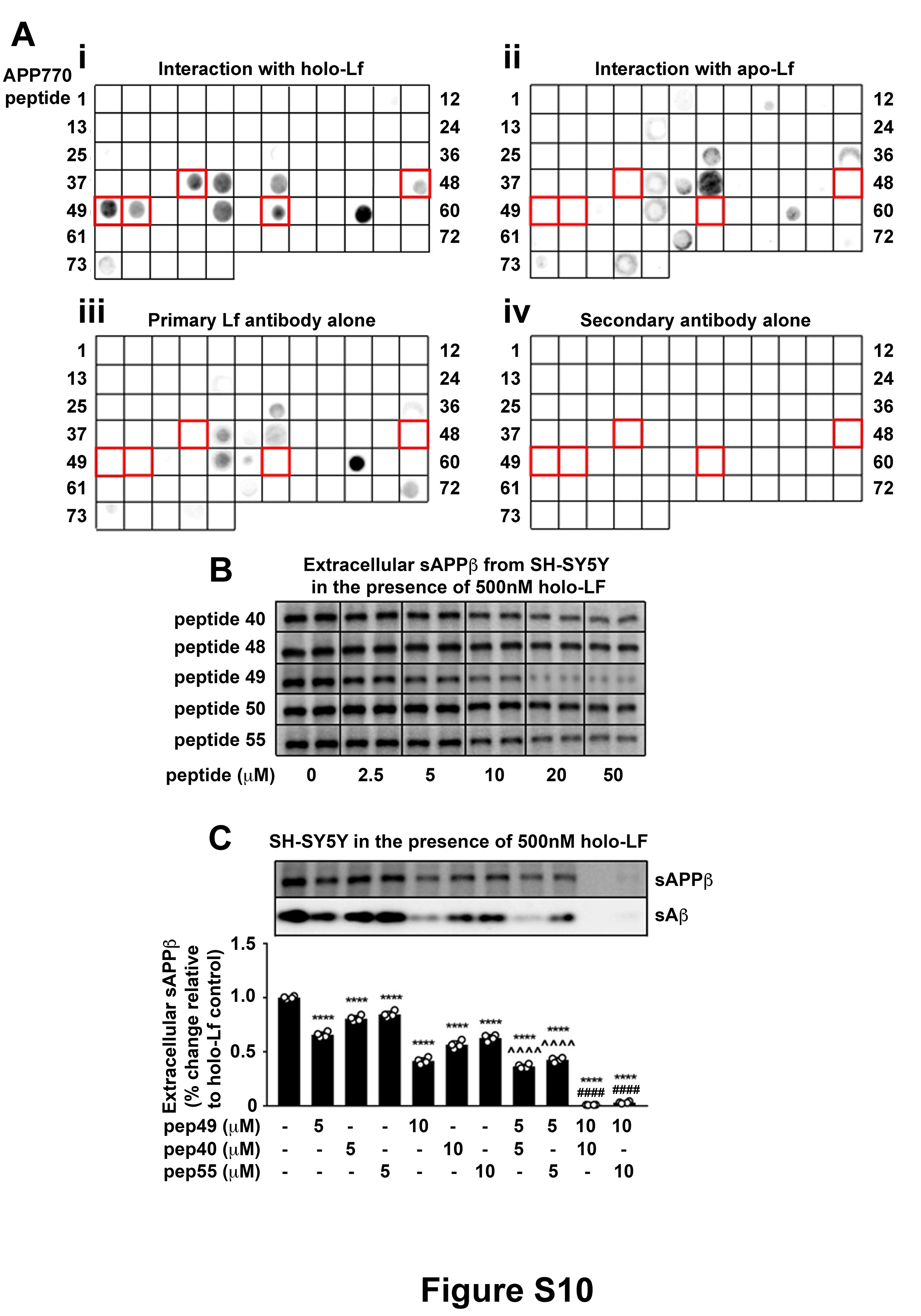

Supplement: Supplementary file 11 — Supplementary Figure 10 [file 41380_2021_1248_MOESM11_ESM.tif]
